# Microfluidic organ-on-chip systems for periodontal research: advances and future directions

**DOI:** 10.3389/fbioe.2024.1490453

**Published:** 2025-01-07

**Authors:** Gopu Sriram, Hardik Makkar

**Affiliations:** ^1^ Faculty of Dentistry, National University of Singapore, Singapore, Singapore; ^2^ Department of Biomedical Engineering, College of Design and Engineering, National University of Singapore, Singapore, Singapore; ^3^ Center for Innovation & Precision Dentistry, School of Dental Medicine and School of Engineering & Applied Sciences, University of Pennsylvania, Philadelphia, PA, United States

**Keywords:** periodontal disease, organ-on-a-chip, microphysiological systems, host-microbe interactions, biofilms, microfluidic systems, *in vitro* models, biofabrication

## Abstract

Advances in tissue engineering and microfluidic technologies have enabled the development of sophisticated *in vitro* models known as organ-on-a-chip (OoC) or microphysiological systems. These systems enable to potential to simulate the dynamic interactions between host tissues and their microenvironment including microbes, biomaterials, mechanical forces, pharmaceutical, and consumer-care products. These fluidic technologies are increasingly being utilized to investigate host-microbe and host-material interactions in oral health and disease. Of interest is their application in understanding periodontal disease, a chronic inflammatory condition marked by the progressive destruction of periodontal tissues, including gingiva, periodontal ligament, and alveolar bone. The pathogenesis of periodontal disease involves a complex interplay between microbial dysbiosis and host immune responses, which can lead to a loss of dental support structures and contribute to systemic conditions such as cardiovascular disease, diabetes, and inflammatory bowel disease. This provides a comprehensive overview of the latest developments in millifluidic and microfluidic systems designed to emulate periodontal host-microbe and host-material interactions. We discuss the critical engineering and biological considerations in designing these platforms, their applications in studying oral biofilms, periodontal tissue responses, and their potential to unravel disease mechanisms and therapeutic targets in periodontal disease.

## 1 Introduction

Periodontitis is a chronic inflammatory disease characterized by the progressive destruction of the tissues that support the teeth, including the gingiva (gums), periodontal ligament, and alveolar bone (Darveau, 2010). This destructive process is initiated by a dysbiotic shift in the subgingival plaque, a complex biofilm that colonizes the periodontal pockets (Bosshardt, 2018; Berezow and Darveau, 2011). While the primary consequence of periodontitis is the loss of dental support structures, it is increasingly recognized that the disease has broader systemic implications. Periodontitis has been associated with various systemic conditions, including cardiovascular disease, diabetes mellitus, and inflammatory bowel disease, suggesting a bidirectional relationship between oral and systemic health (Darveau, 2010; Hajishengallis, 2015; Seneviratne et al., 2020). Understanding the mechanisms that underpin these associations is critical, not only for improving periodontal treatment outcomes but also for mitigating systemic health risks.

**FIGURE 1:**
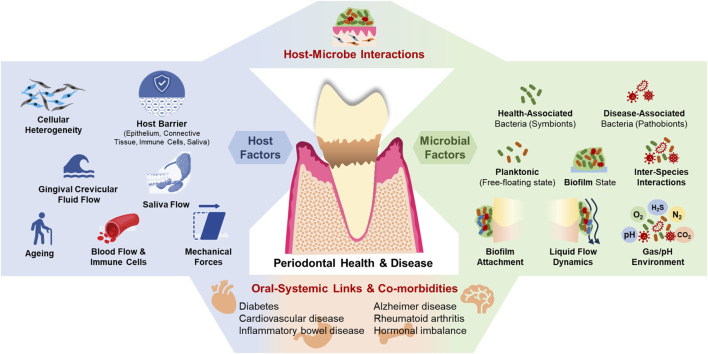
The periodontal microenvironment, its interaction with microbes, and systemic co-morbidities.

The pathogenesis of periodontitis is multifactorial, involving a complex interplay between microbial factors and host immune responses (Lamont et al., 2023; Marsh and Zaura, 2017). The oral cavity hosts a diverse microbial community, that normally exists in a symbiotic relationship with the host (Figure 1). In a healthy state, this microbiota contributes to homeostasis by regulating innate immunity and enhancing mucosal barrier function (Devine et al., 2015; Buskermolen et al., 2018; Shang et al., 2018; Shang et al., 2019). Periodontitis involves changes in the microbial species composition of subgingival plaque, with a decrease in health-associated species and an increase in disease-associated species. As periodontitis progresses, the oral microbiota shifts from being predominantly composed of gram-positive aerobes to one dominated by gram-negative anaerobes. This transition toward oral dysbiosis is believed to occur over a long period, gradually altering the host-microbe relationship from a balanced, symbiotic one to a pathogenic one. As this process unfolds, the host’s oral health deteriorates, eventually leading to clinical disease. At the same time, distinct microbial complexes emerge. The first such complex linked to disease is the “orange complex”, made up of gram-negative, anaerobic bacteria like *Prevotella intermedia* and *Fusobacterium nucleatum*. As the disease advances, the microbiota shifts to the “red complex”, which includes the periodontal pathogens *Porphyromonas gingivalis*, *Tannerella forsythia*, and *Treponema denticola* (Darveau, 2010; Hajishengallis, 2015; Abusleme et al., 2021; Preshaw et al., 2012; Sanz et al., 2020). This transition from health to disease involves not only changes in microbial composition but also alterations in the local microenvironment, including shifts in pH, oxygen levels, nutrient availability, and evasion of local innate immune responses, which favor the growth of pathogenic species (Pollanen et al., 2013). Key microbial factors involved in the pathological mechanisms include inter-bacterial interactions (Lamont et al., 2023; Jakubovics, 2015), biofilm matrix (Jakubovics et al., 2021), microbial adhesion to host tissues (Sterzenbach et al., 2020), the fluid environment (both liquid and gas) within periodontal tissues (Pollanen et al., 2013; Mark Welch et al., 2016), and the interaction between host cells and microorganisms (Lamont et al., 2023; Pan et al., 2019). Systemic dissemination of bacteria, their byproducts, and inflammatory cytokines further complicates the disease progression (Hajishengallis, 2022; Hajishengallis and Chavakis, 2021).

The host’s response to microbial dysbiosis is equally complex. The gingival epithelium serves as the first line of defense, providing a physical barrier that limits microbial invasion (Schroeder and Listgarten, 1997). Beneath this epithelial layer lies the connective tissue, which contains a rich network of fibroblasts, immune cells, and extracellular matrix components that actively participate in the immune response (Fournier et al., 2010). The connective tissue matrix, along with the blood vessels within it, serves as additional barriers and protects the host from microbes (Fournier et al., 2010; Wang and Somerman, 1991; Embery et al., 2000). The innate immune response is finely tuned and regulated by a multitude of factors such as fibroblast heterogeneity, dynamics of gingival crevicular fluid (GCF) flow, and microbial factors. Other modulators include aging, systemic diseases, hormonal fluctuations, dietary factors, medications, and lifestyle behaviors such as smoking and alcohol consumption (Pollanen et al., 2013; Pan et al., 2019; Taylor and Preshaw, 2016). Together, these factors contribute to the dynamic environment of the periodontal tissues, where continuous interactions between the host and the microbiome determine the state of health or disease. Hence, to accurately model host-microbe interactions *in vitro*, it is essential to replicate native host tissue and microbial features such as barrier function, GCF and saliva flow, shear stress, surface stiffness, cell-cell, cell-matrix, and cell-microbe interactions (Tabatabaei et al., 2020; Barrila et al., 2018).

Advances in tissue engineering and microfluidic technologies have paved the way for next-generation *in vitro* models that better simulate the dynamic interactions between host and microbes in periodontitis (Aveic et al., 2021; Sanchez et al., 2021; Wang et al., 2024). By incorporating various microphysiological features, 3D organotypic cultures and organ-on-a-chip (OoC) systems offer a physiologically relevant platform to study the intricate host-microbe interactions in periodontal health and disease. The application of OoC technologies to emulate the microphysiological and microenvironmental parameters of various tissues and organ systems is gaining tremendous interest (Shoji et al., 2023) and contributing to the change in the regulatory landscape (Stewart et al., 2023). OoC systems emulating barrier tissues such as skin-on-chip, lung-on-chip, and gut-on-chip have demonstrated the enormous potential to mimic various microphysiological interactions between host barrier tissues, microbes, materials, and environmental factors (Zheng et al., 2016; Ahadian et al., 2018; Kim and Sung, 2024; Ugodnikov et al., 2024). Following this trend, various millifluidic and microfluidic-based systems have been developed and utilized to emulate dental, oral, and periodontal tissues (Huang C. et al., 2023; Ardila et al., 2023) and their microphysiological interactions with microbes and biomaterials in health and diseased states (Makkar et al., 2023a; Franca et al., 2020; Rodrigues et al., 2021; Adelfio et al., 2023; Jin et al., 2022; Lee et al., 2023; Muniraj et al., 2023; Rahimi et al., 2018).

In this review, we explore the advances in millifluidic and microfluidic systems designed to mimic periodontal host-microbe and host-material interactions. We discuss key aspects related to engineering and biological considerations towards the design of these fluidic OoC platforms and recapitulation of the complex, dynamic microenvironment of the oral biofilms, gingival/periodontal tissues, and their interactions. Further, we provide an overview of the application of these fluidic systems in oral biofilm studies, host-microbe, and host-material interactions in the context of periodontal health and disease.

## 2 Modeling periodontal host-microbe interactions

Modeling the complex interactions between the host and microbiome in periodontitis is a significant challenge due to the multitude of factors involved. The initiation and progression of periodontitis are linked to shifts in the oral microbial community, and the ensuing immune responses triggered by microbial dysbiosis lead to severe destruction of periodontal tissues (Bosshardt, 2018; Berezow and Darveau, 2011). This complex interplay between microbial-microbial and host-microbial interactions is difficult to capture in experimental models, yet it is essential for unraveling the molecular mechanisms underlying the disease and for developing new therapeutic strategies. *In vivo* models, such as those using animals like rodents, dogs, and nonhuman primates, have long been considered the gold standard for studying periodontitis (Kantarci et al., 2015; Schwarz et al., 2015). These models offer several advantages, including the ability to simulate the full spectrum of periodontitis progression, from initial microbial colonization to advanced tissue destruction, providing insights into the complex host-microbe interactions and disease dynamics (Kantarci et al., 2015; Graves et al., 2008). Additionally, animal models can reflect the complex microbiological characteristics of subgingival plaque in different states representative of health, gingivitis, or periodontitis. In addition to studying disease progression, animal models also allow for the examination of healing processes in periodontal and peri-implant tissues, offering critical insights into the effects of various biological materials and regenerative strategies (Kantarci et al., 2015; Graves et al., 2008). However, the physiological and anatomical differences between animal models and humans, such as differences in the composition of plaque biofilm and saliva, flow rate, continuously erupting incisors in rodents and other factors limit the translatability of findings directly to human relevance. Small animal models, while useful, often lack the complexity of human dentition, which limits their applicability to human disease (Kantarci et al., 2015). Furthermore, the intricate biology of these models makes it difficult to isolate and analyze individual factors systematically. Beyond these scientific challenges, ethical concerns, high costs, and lengthy timelines associated with animal research have prompted the development of alternative models.

*In vitro* models for studying periodontal host-microbe interactions have traditionally relied on monolayer culture systems, where planktonic bacteria are added to monolayers of host cells using the principle of multiplicity of infection (MOI) (Lamont et al., 1995). These models evaluate dose- and time-dependent interactions between host cells and microbes, are simple, cost-effective, enable high-throughput screening, and provide the potential to dissect specific factors and pathways involved in periodontal disease (Tabatabaei et al., 2020; Mountcastle et al., 2020). However, these models are reductionistic, limited by their inability to replicate the complex architecture and dynamic microenvironment of native gingival and periodontal tissues. The interactions in these systems are often short-lived (4–24 h) (Bao et al., 2015a; Bostanci et al., 2015; Pinnock et al., 2014) due to the toxic by-products from microbial metabolism (Mempel et al., 2002; Wiegand et al., 2009). To mitigate these challenges, attenuated bacteria or bacterial surrogates, such as Toll-like receptor agonists or virulence factors like lipopolysaccharide (LPS), are often used (Sriram et al., 2016; Engen et al., 2017). These challenges have driven a shift towards the development and adoption of three-dimensional (3D) organotypic culture models that aim to closely emulate the native tissue microenvironment and architecture.

3D organotypic culture models range from epithelium or connective tissue-only constructs to more complex full-thickness tissue equivalents, incorporating both epithelial and connective tissue components. Through the incorporation of stratified layers of multiple cell types and extracellular matrix components, 3D cultures mimic the native tissue architecture and barrier properties of epithelium and connective tissue matrix of gingival and periodontal tissues (Buskermolen et al., 2018; Shang et al., 2018; Shang et al., 2019; Dongari-Bagtzoglou and Kashleva, 2006; Dabija-Wolter et al., 2012; Belibasakis et al., 2013; Makkar et al., 2023b; Bao et al., 2015b). These 3D organotypic culture models have been increasingly utilized for host-biomaterial (Chai et al., 2012; Moharamzadeh et al., 2008; Hu et al., 2022; Klausner et al., 2007), implant-soft tissue (Chai et al., 2012; Mikolai et al., 2020; Sakulpaptong et al., 2022), and host-microbe (Buskermolen et al., 2018; Shang et al., 2018; Shang et al., 2019; Bostanci et al., 2015; Pinnock et al., 2014; Belibasakis et al., 2013; Makkar et al., 2023b; Cavalcanti et al., 2015; Yadev et al., 2011) interaction studies. For host-microbe studies, bacteria can be introduced as either planktonic cells or pre-formed biofilms based on colony-forming units (CFU) per milliliter or surface area of the construct (Buskermolen et al., 2018; Shang et al., 2018; Shang et al., 2019; Makkar et al., 2023b; Ingendoh-Tsakmakidis et al., 2019; Makkar et al., 2022). Alternatively, biofilms can be cultivated on various substrates, including coverslips, hydroxyapatite discs, enamel slices, or implant surfaces. These biofilms can then be directly applied to the surface of the organotypic cultures, simulating the host-microbial interface observed *in vivo* (Thurnheer et al., 2014). Using these exposure strategies, 3D organotypic cultures have been used to study a wide range of phenomena, including microbial adhesion, biofilm formation, invasion, impact on epithelial barrier integrity, immune cell recruitment, cytokine production, and overall tissue-level inflammatory responses (Buskermolen et al., 2018; Shang et al., 2018; Shang et al., 2019; Makkar et al., 2023b; Ingendoh-Tsakmakidis et al., 2019; Makkar et al., 2022). Further, 3D organotypic cultures enable the study of both mono-species and multi-species biofilms, providing insights into the complex dynamics of bacterial interspecies interactions and their collective impact on host tissues (Buskermolen et al., 2018; Shang et al., 2018; Shang et al., 2019). This is particularly important for understanding the transition from a healthy, symbiotic microbiome to dysbiotic state associated with periodontal diseases. The ability to mimic these processes *in vitro*, under controlled conditions, provides a valuable tool for investigating the mechanisms underlying periodontal disease pathogenesis and for developing potential therapeutic interventions. Despite these advantages, 3D organotypic models do not fully capture the dynamic aspects of the periodontal microenvironment, such as fluid flow, shear stress, aerobic-anaerobic interface, pH changes, and other host and microbial factors, which play critical roles in shaping host-microbe interactions. To overcome these limitations, there is a growing need to develop fluidic OoC systems that can better emulate the dynamic microenvironment of the oral cavity and offer real-time monitoring of host-microbe interactions.

## 3 Engineering fluidic platforms for periodontal host-microbe and material interactions

**FIGURE 2:**
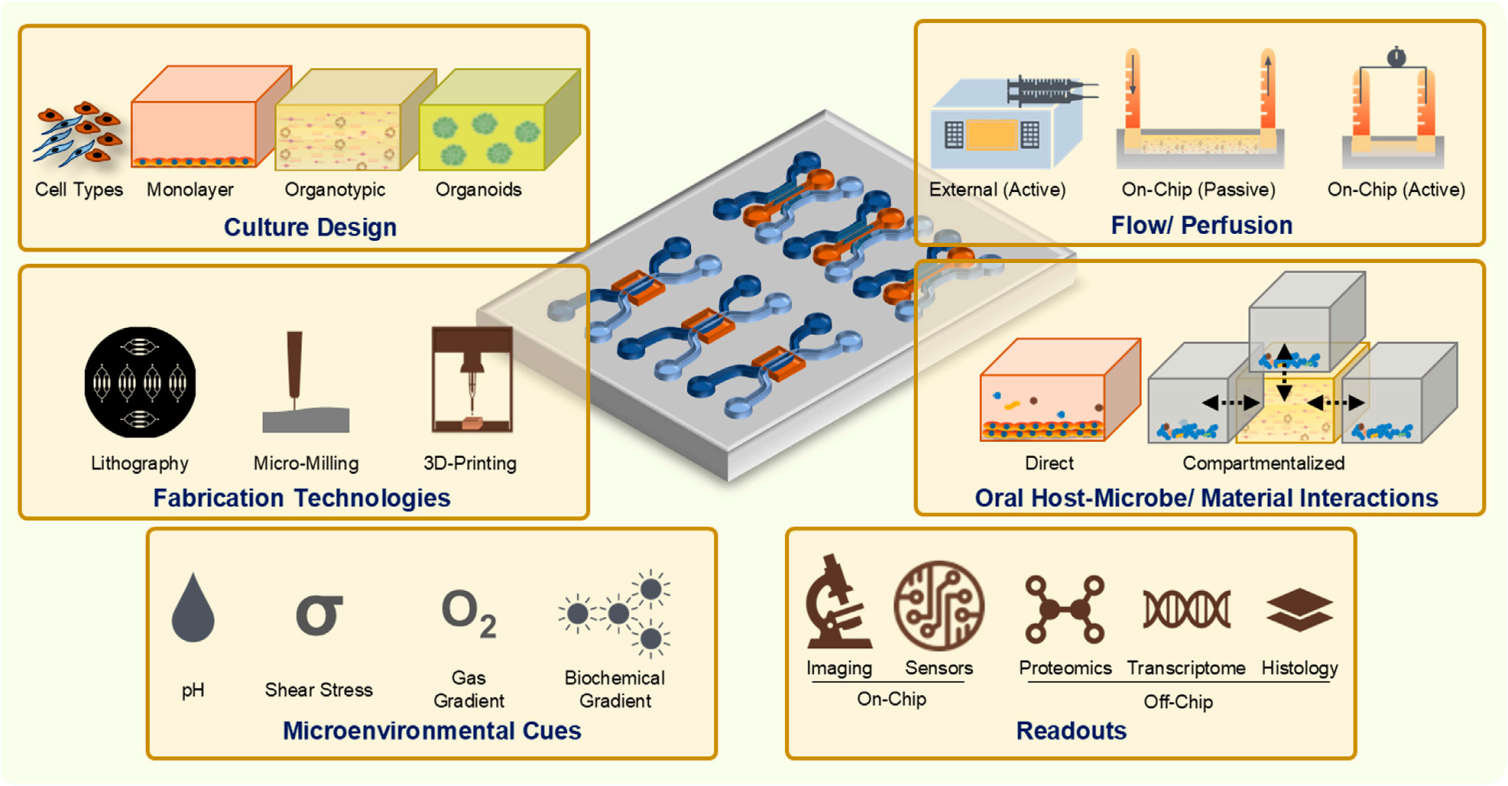
Guiding principles and considerations for the design and development of OoC systems to study periodontal host-microbe and material interactions.

Microfluidic and millifluidic OoC systems have emerged as innovative tools in the field of tissue engineering and bioengineering, offering sophisticated platforms to emulate the complex microenvironments of human tissues (Figure 2). These OoC systems integrate advanced features such as precise geometrical confinement, controlled fluid dynamics, cell patterning, and microenvironmental regulation, which are crucial for replicating the native conditions of human tissues and organs (Zheng et al., 2016; Ahadian et al., 2018; Bhatia and Ingber, 2014; Mou et al., 2022). By incorporating microchannels and microchambers, these systems enable the manipulation of fluid flow, nutrient distribution, waste removal, and collection of cellular secretions, closely mimicking the vascular and interstitial fluid dynamics found *in vivo* (Bhatia and Ingber, 2014; Zhang et al., 2018; Lee and Sung, 2018; Ingber, 2016). These enabling features have led to the application of OoC technologies to model various organ systems and barrier tissues such as the kidneys, liver, bone, lungs, gut, blood vessels, and skin (Shoji et al., 2023; Kim and Sung, 2024; Ugodnikov et al., 2024; Huh et al., 2013; Sutterby et al., 2020; Izadifar et al., 2022).

The ability to spatially compartmentalize cellular, microbial, material, and media components along with the precise control over fluid flow within these devices is crucial for replicating the interactions at tissue-biomaterial and tissue-microbe interfaces, particularly in barrier tissues such as the tooth, gingiva, and oral mucosa. This capability provides opportunities to study the intricate dynamics of microbial colonization, biofilm formation, and host immune responses under conditions that closely resemble the native oral environment than traditional static culture systems. Consequently, there is a growing trend towards the use of microfluidic OoC and microphysiological systems to model oral and dental barrier tissues *in vitro* (Huang C. et al., 2023; Ardila et al., 2023; Franca et al., 2022; Dasgupta et al., 2024). These OoC models encompass a variety of platforms including tooth-on-chip (Franca et al., 2020; Rodrigues et al., 2021; Hu et al., 2022), gingiva-on-chip (Jin et al., 2022; Lee et al., 2023; Muniraj et al., 2023; Gard et al., 2023), gingival crevice-on-chip (Makkar et al., 2023a), oral mucosa-on-chip (Rahimi et al., 2018; Ly et al., 2021), oral mucositis-on-chip (Ly et al., 2022; Ly et al., 2024), oral cancer-on-chip (da Costa Sousa et al., 2024), pulp-like tissues on-chip (Zhang et al., 2022), and salivary gland-on-chip (Song et al., 2021), each designed to replicate specific features of dental and oral health and disease.

Fluidic systems have been used to model the colonization and maturation of complex oral biofilms (Rath et al., 2017; Luo et al., 2019; Kristensen et al., 2020; Gashti et al., 2016), which pave the way towards understanding the responses of these microbial communities to therapeutic agents or interventions under dynamic flow conditions. This capability is particularly valuable for testing the efficacy of therapeutic agents and oral-care formulations in a controlled, yet physiologically relevant setting. These systems have also facilitated investigations into host-microbe-material interactions, providing insights into the innate immune response and tissue barrier functions (Makkar et al., 2023a; Adelfio et al., 2023; Jin et al., 2022; Lee et al., 2023; Muniraj et al., 2023; Rahimi et al., 2018; Ly et al., 2021). Furthermore, these systems facilitate the study of fluid dynamics within the oral cavity, including the flow of saliva, GCF, interstitial fluid, and oral-care formulations, as well as the mechanical forces that influence tissue behavior and microbial interactions (Makkar et al., 2023a; Adelfio et al., 2023; Lee et al., 2023; Muniraj et al., 2023; Mishra et al., 2023). The ability to simulate these conditions *in vitro* offers significant insights into the factors that contribute to oral health and disease, including the mechanical and biochemical interactions that occur within the oral microenvironment. In addition to studying host-microbe interactions, microfluidic and millifluidic OoC systems have also been applied to evaluate the biocompatibility and performance of dental materials and implants (Franca et al., 2020; Rahimi et al., 2018; Hu et al., 2022; Ly et al., 2021; Koning et al., 2021). By mimicking the barrier, mechanical and fluid flow conditions of oral tissues, these systems have provided insights on assessment of biocompatibility, cellular viability, and tissue-level responses. Overall, microfluidic and millifluidic-based culture systems are revolutionizing our understanding of host-microbe and host-material interactions, offering unprecedented opportunities to study the complexities of oral health and disease in a controlled, physiologically relevant manner.

### 3.1 Design considerations

The design characteristics of an OoC system determine its functionality, applications, and potential limitations (Figure 2). Customization enables the appropriate replication of the intended microphysiological parameters of the tissues and their interface with the internal and external milieu. Fluidic devices with varied designs have been developed to study oral biofilms, dental/oral tissues, and their interactions with biomaterials and microbes (Huang C. et al., 2023). The design features of an OoC system hinge on the specific functionalities required to accurately model physiological processes (Cao et al., 2023). The complexity of the system should be minimized to represent the biological application without adding unnecessary elements that complicate use and analysis. A critical aspect of this design process involves selecting the approach for constructing functional tissues within the OoC. In a top-down approach, primary tissues such as slices from biopsies or dentin slices from teeth are integrated into the OoC system (Franca et al., 2020; Rodrigues et al., 2021; Hu et al., 2022). Similarly, tissue equivalents engineered using 3D organotypic cultures can be integrated into the OoC system in a top-down approach. Conversely, in a bottom-up approach, isolated cells from primary sources, immortalized lines, or stem cells are cultured within an initially empty microfluidic environment, which supports their remodeling into functional neo-tissues. In this strategy, the cells may be cultured as monolayers (Rodrigues et al., 2021; Jin et al., 2022; Hu et al., 2022), spheroids (Mishra et al., 2023), or incorporated within hydrogels (Makkar et al., 2023a; Muniraj et al., 2023; Ly et al., 2024; Zhang et al., 2022) that provide a 3D architecture. The chosen strategy influences the overall design of the OoC architecture, serving both to organize and support cells in a specific configuration and to route fluids, such as culture medium, to mimic the *in vivo* connectivity between tissue components.

Under static culture conditions, tissues thicker than 400 μm encounter limitations in the diffusion of oxygen and nutrients, hindering the distribution of essential resources throughout the entire tissue (Li et al., 2016). This limitation also leads to the rapid accumulation of toxic cellular metabolites in the surrounding media. Consequently, tissue thickness is directly correlated with tissue health in static culture settings. Fluidic OoC systems address these challenges through the incorporation of design elements that enable continuous perfusion, allowing active media flow below, over and/or through the tissue, and enhancing the transport of nutrients and waste products (Barrila et al., 2018; Zhang et al., 2018). Microfluidic perfusion systems offer precise control over nutrient delivery rates and can establish a stable, well-defined microenvironment. For instance, the nutrients can be delivered under constant flow rate for cellular maintenance, or in a cyclical manner to mimic time-dependent physiological processes (Ahadian et al., 2018; Leung et al., 2022). These perfusion systems combined with optimal design of the cellular chambers and media channels, provide controlled media flow that facilitates deeper perfusion into tissues, maintains nutrient supply, and effectively drain the metabolic wastes. In terms of design, it is also crucial to consider the geometry of the tissue. While monolayer cultures can be perfused efficiently within straight microfluidic channels, this is not always practical for tissue-engineered 3D tissue equivalents and *ex vivo* tissue samples. Optimizing the design elements using insights from computational fluid dynamics can help model and predict flow patterns, shear forces, and mass transport parameters relevant to the intended application. Focused reviews are available that discuss these strategies and design (Zheng et al., 2016; Cao et al., 2023; Leung et al., 2022; McLean et al., 2018).

Although OoC devices exhibit diverse architectural variations, they can generally be categorized into two types. The first type involves culturing cells as 3D tissues that interact within a controlled environment, often using micropillars or microwell arrays to maintain tissue structure and compartmentalization (Makkar et al., 2023a; Franca et al., 2020; Rodrigues et al., 2021; Rahimi et al., 2018; Ly et al., 2024; Zhang et al., 2022). The second type is designed to support the formation of natural barriers between fluid compartments (Muniraj et al., 2023; Sriram et al., 2018). This later architecture is particularly suited for tissues that naturally exist at an air-liquid interface, such as the oral mucosa, gingiva, gut, lung, and skin. This configuration allows the stratification, differentiation, and formation of the epithelial barrier, and enables the study of selective transport processes across the barrier tissue. Microphysiological systems that recapitulate dental, oral mucosal, and gingival barrier tissues generally employ a design where a culture chamber is positioned between two parallel channels. The arrangement of microchannels within these devices, whether in horizontal or vertical stacking, presents distinct opportunities and limitations. Horizontally-stacked channels facilitate compartmentalization and allow real-time visualization of cells, extracellular matrices, and bacteria, as well as their responses to dental materials and microorganisms (Makkar et al., 2023a; Franca et al., 2020; Rodrigues et al., 2021; Rahimi et al., 2018; Ly et al., 2024). However, this configuration may not provide the necessary air-liquid interface required for the stratification and differentiation of keratinocytes and the development of epithelial barrier functions. In contrast, vertically-stacked designs better mimic the oral cavity and support the formation of a protective barrier by providing the necessary air-liquid interface for efficient keratinocyte stratification and differentiation (Muniraj et al., 2023; Sriram et al., 2018). Ultimately, the design architecture and culture strategy must be carefully chosen to align with the intended functionality of the OoC, ensuring that the system effectively replicates the desired physiological conditions.

### 3.2 Material selection and fabrication

The choice of materials to fabricate the fluidic systems is guided by multiple factors, including the desired functionality of the device, the methods available for microfabrication, the type of readouts required, and the need for biocompatibility. Frequently utilized materials include poly (dimethylsiloxane) (PDMS), glass, and various thermoplastics such as poly (methyl methacrylate), polycarbonate, and cyclic olefin copolymer (Leung et al., 2022). Each of these materials presents unique benefits and drawbacks, requiring a careful balance between functionality and feasibility of the fabrication process (Campbell et al., 2021) (Table 1).

**TABLE 1:** Materials and techniques used to fabricate organ-on-chip devices for oral host-microbe interaction.

Material	Advantages	Limitations	Fabrication techniques
PDMS (Makkar et al., 2023a; Rodrigues et al., 2021; Jin et al., 2022; Lee et al., 2023; Rahimi et al., 2018; Eun and Weibel, 2009; Kim and Ingber, 2013)	• Biocompatible • Gas Impermeable • Optical Transparency • Elastic Properties • Nano/micro-scale features	• Protein Adsorption • Scaling up for mass production	• Soft Lithography
Thermoplastics (Franca et al., 2020; Muniraj et al., 2023; Gard et al., 2023; Rath et al., 2017; Mishra et al., 2023; Lam et al., 2016; Nance et al., 2013)	• Inert (pre-polymerized) • Optically transparent • Mass production • Low-cost • Gas Impermeable	• Autofluorescence • Rigidity • Difficulty in machining complex structure	• Laser cutting • Micro-milling • 3D Printing • Injection molding • Thermal Bonding
3D printing (light curable resins) (Adelfio et al., 2023; Kristensen et al., 2020)	• Tunable mechanical properties • Low-cost • Design freedom	• Autofluorescence • Optically translucent • Post processing • Toxicity • Surface roughness	• Digital Light processing printing • Sterolithography • Extrusion printing
Glass	• Inert • Optically transparent • Mass production • Gas Impermeable • Biocompatible	• Expensive fabrication • Fragile • Biologically non receptive surface	• Laser cutting • Micro-milling

PDMS, is the most prevalent material in OoC systems applied to dental research (Makkar et al., 2023a; Franca et al., 2020; Rodrigues et al., 2021; Rahimi et al., 2018; Ly et al., 2022), owing to its versatility in creating high-resolution micro- and nanostructures via soft lithography. Its biocompatibility, optical transparency, and gas permeability make it appropriate for biological applications and on-chip imaging. The elastic properties of PDMS also allows for the application of mechanical forces to cultured cells and ECM (Lee et al., 2023). Despite these advantages, PDMS tends to adsorb and absorb various (bio)chemicals, which can potentially influence experimental outcomes (Halldorsson et al., 2015). Additionally, the inherent hydrophobicity of PDMS can hamper the attachment of cells or ECM, necessitating surface modification to enhance hydrophilicity (various strategies have been reviewed thoroughly elsewhere (Sutthiwanjampa et al., 2023; Neves et al., 2024). Oxidation, commonly achieved through plasma treatment, is a common method for increasing the hydrophilicity of PDMS. However, this process can increase its stiffness, rendering it unsuitable for applications requiring precise control over mechanical properties. Silanization with 3-aminopropyl triethoxysilane (APTES) can be supplemented with oxidation to maintain long-term hydrophilicity. This treatment introduces amine functional groups on the PDMS surface, allowing it to act as a coupling agent for subsequent coatings. Recently, polydopamine coatings have been explored as a promising approach to enhance cellular and matrix adhesion on PDMS surfaces. This method offers the dual benefits of improving the adhesion properties and mitigating the contraction and detachment issues associated with matrices like collagen (Park et al., 2019).

Glass is a durable, inert material that offers excellent optical clarity, making it ideal for high-resolution imaging. However, it is expensive and requires specialized processing capabilities. Thermoplastic materials such as poly (methyl methacrylate) and polycarbonate are also used to fabricate OoC devices for dental applications. These materials are known for their gas impermeability and transparency, and they offer versatility in fabrication techniques, such as 3D printing (Kristensen et al., 2020), thermal bonding of pre-polymerized sheets (Muniraj et al., 2023; Hu et al., 2022), or mass production *via* injection molding (Zhang et al., 2022). A significant advantage of thermoplastics is their suitability for scaling up production and enabling high-throughput readout capabilities.

In the context of periodontal disease, the selection of materials for OoC systems plays a critical role in replicating the complex host-microbe interactions. The surface properties of the materials, such as roughness and hydrophobicity, significantly influence microbial adhesion and biofilm formation, which are key aspects of periodontal disease progression. Materials with tailored surface characteristics can either promote or inhibit microbial colonization, allowing for the controlled study of biofilm dynamics and host immune responses. Moreover, the gas permeability of materials is a crucial consideration when modeling the unique microenvironments of the oral cavity, particularly the aerobic and anaerobic conditions found in subgingival pockets. The gas impermeability of thermoplastics can be beneficial for the culture of anaerobic periodontal bacteria, whereas the gas permeability of PDMS could be utilized for the culture of aerobic bacteria and host cells. Optimal use of the material properties could be utilized to simulate the oxygen gradients present in gingival sulcus and periodontal pockets, and hence, replicate the microbial behavior and host tissue responses. Furthermore, the mechanical properties of materials, especially the elasticity of PDMS, enable the application of physiological mechanical forces (Lee et al., 2023), such as those experienced during mastication. This mechanical stimulation can affect both host tissue responses and microbial dynamics, providing a more physiological representation of the oral environment and its influence on periodontal disease progression.

### 3.3 Fluidic control

Effective fluid control is fundamental in OoC systems to accurately replicate the dynamic microphysiological conditions of human tissues (Cao et al., 2023). Effective management of fluid flow is crucial for simulating periodontal microenvironments, impacting microbial dynamics, biofilm development, and host-microbe interactions. Different methods are employed to manage fluid flow through these devices, each offering unique benefits and limitations depending on the experimental objectives which have been reviewed thoroughly elsewhere (Leung et al., 2022; Byun et al., 2014).

Fluid flow within OoC systems is typically managed by external pumps such as peristaltic, syringe, and pneumatic pumps (Table 2) (Makkar et al., 2023a; Adelfio et al., 2023; Muniraj et al., 2023; Rahimi et al., 2018; Mishra et al., 2023; Koning et al., 2021). These pumps offer precise regulation of the fluid flow rate and direction (Muniraj et al., 2023; Mishra et al., 2023). However, their operation can be cumbersome owing to complex connections with multiple tubings, that can increase the risk of contamination and bubble formation, potentially limiting their broader adoption. Alternatively, rocker platforms, hydrostatic pressure, or tension-driven pumpless designs (Makkar et al., 2023a; Franca et al., 2020; Rodrigues et al., 2021; Rahimi et al., 2018), offer simplified fluid control without the need for complex setups. These methods are user-friendly, and do not demand specialized expertise. However, the fluid flow in rocker platforms is bidirectional, while those based on gravity or hydrostatic pressure have variable or decreasing flow rates over time (Makkar et al., 2023a), which can limit precise control of fluid flow and restrict their application.

**TABLE 2:** Major fluid pumping techniques used in organ-on-chip systems to study oral host-microbe interaction.

Flow principle	Flow pattern	Flow principle	Advantages
Gravity or Hydrostatic (Makkar et al., 2023a)	Continuous (Decreasing or Constant)	Passive (hydrostatic, or gravity-driven flow)	• Easy assembly • Low cost • Elimination of connectors/tubing
Syringe (Lee et al., 2023)	Continuous	Active	• Very low flow rates • Ability to add more than one media type (cell culture media, bacterial broth) • High throughput with the use of channel splitters for cell and bacterial seeding
Peristaltic (Adelfio et al., 2023; Muniraj et al., 2023; Gard et al., 2023; Rath et al., 2017; Mishra et al., 2023; Lam et al., 2016)	Continuous	Active	• Simulates pulsatile flow • High throughput • Enables recirculation • Can be pneumatically controlled • Custom programmable
Pneumatic (Gard et al., 2023)	Continuous	Active	• Can be miniaturized for on-chip application • Enables recirculation • Controlled flow rates *via* setting of stroke frequency • High throughput

Beyond pumps, other key components such as flow splitters, valves, and gradient generators play a vital role in ensuring precise fluid management and experimental versatility (Chen et al., 2020). Flow splitting is essential for applications requiring multiplexing or the creation of differential biochemical environments. By dividing a single fluid stream into multiple paths, researchers can simulate varied conditions across different regions of the chip, which is crucial for studies involving heterogeneous cell populations or variable microbial environments (Ainscough et al., 2022). This capability enables the examination of complex interactions under controlled, varied conditions. Further, incorporating valves into the fluidic path of OoC systems offers significant flexibility. Valves facilitate the switching of operations between stages such as cell or bacterial seeding, perfusion, culture, and downstream collection of cellular byproducts and biomarkers for analysis (Muniraj et al., 2023). They enable precise control over media exchange, preventing cross-contamination and allowing for sequential processing. This functionality is particularly important for experiments requiring multiple phases or for time-course studies (Huang S. et al., 2023).

Advancements in micro-pump and micro-valve technologies have led to the integration of pumps and valves directly into the chip (Gard et al., 2023; Azizgolshani et al., 2021; Ong et al., 2019). Incorporating the pumps and valves directly into the chip provides an integrated and automated approach to fluid management. These on-chip pumps and valves offer precise flow control and are better suited for continuous long-term studies and high-throughput experiments. These technologies eliminate the need for complex external setups, providing an integrated solution for fluid control. However, the design and fabrication of these integrated systems can be expensive and technically demanding requiring specialized knowledge and resources that may not be readily available in all research labs.

Simulating pulsatile flow is essential for replicating the physiological blood and interstitial fluid flow dynamics experienced in periodontal tissues. This approach provides a more realistic simulation of the mechanical forces experienced by periodontal tissues (Muniraj et al., 2023; Mishra et al., 2023). Pulsatile flow can be achieved through the peristaltic action of external pumps, which mimic the natural rhythmic contraction and relaxation cycles of fluid movement. Alternatively, advanced on-chip pumps with integrated micro-valves can be programmed to produce a controlled pulsatile flow, offering a compact and precise solution for simulating these dynamics within organ-on-chip systems. Pulsatile flow can influence microbial behavior, biofilm development, and immune cell migration, making it an essential consideration for studies focused on the dynamic interactions within periodontal tissues.

### 3.4 Sterilization

Ensuring sterility of OoC devices is crucial to prevent microbial contamination, particularly given the complexity of their microfluidic components. The diverse range of materials used in these devices necessitates careful selection of sterilization methods to avoid damage that could lead to leaks or compromised functionality. PDMS and glass-based devices are generally amenable to autoclave sterilization. Thermoplastics like poly (methyl methacrylate) and polycarbonate are susceptible to heat damage, making traditional autoclave sterilization unsuitable. Instead, alternative sterilization techniques, such as UV, x-ray, or ethanol treatment, are often used in laboratory settings. However, these methods come with limitations: UV light may not effectively penetrate certain materials, while ethanol can cause partial dissolution of PMMA and may be absorbed by PDMS, potentially leaching out and affecting cell cultures. For more robust sterilization, gamma irradiation and ethylene oxide treatment are recommended, as they offer effective sterilization without compromising the integrity of the device.

### 3.5 Cellular considerations

One of the crucial considerations for developing OoC systems is the selection of relevant cells and its sources. The OoC platforms have the advantage of integrating a range of human-derived cell lines and maintaining the optimum microenvironment required for their successful culture. The various cell types that can be incorporated into these systems range from primary cells (derived from healthy and diseased sources), immortalized cell lines, stem cells, and their differentiated progeny.

Primary cells offer the major advantage of being phenotypically similar to cells observed under *in vivo* conditions, potential to derive health and disease-associated phenotypes, and towards personalized medicine (Beigel et al., 2008). Use of primary cells is a popular choice for dental (Rodrigues et al., 2021) and periodontal (Makkar et al., 2023a; Adelfio et al., 2023; Jin et al., 2022; Gard et al., 2023; Ly et al., 2024) OoC platforms. However, the use of primary cells is not without challenges, as these cells often undergo post-mitotic changes and senescence *in vitro*, which limits their viability for large-scale culture and tissue engineering purposes. Alternatively, immortalized cell lines and cancer cells are potential alternatives to primary cells especially in the early development of OoC barrier systems (Lee et al., 2023; Muniraj et al., 2023; Rahimi et al., 2018). An added advantage of human telomerase reverse transcriptase (hTERT) immortalized cells is their ability to combine the physiological traits of primary cells with an extended *in vitro* culture lifespan (Muniraj et al., 2023; Buskermolen et al., 2016). These cells exhibit stable karyotype and can be induced to differentiate, exhibiting tissue-specific features and expressing markers indicative of differentiation (Muniraj et al., 2023; Buskermolen et al., 2016; Hare et al., 2016; Toouli et al., 2002). In the context of oral mucosal tissue engineering, hTERT immortalized keratinocytes from gingiva (Buskermolen et al., 2018; Buskermolen et al., 2016) and floor of the mouth (Muniraj et al., 2023; Hu et al., 2022) as well as gingival fibroblasts (Buskermolen et al., 2018) have been successfully used to develop organotypic cultures of oral mucosa and gingiva and their application in host-material and microbiome interaction studies. These models have demonstrated the capacity to generate multilayered epithelial structures that express key differentiation markers and antimicrobial peptides, closely resembling native tissue.

Stem cells (embryonic, adult, and induced pluripotent) have the property of self-renewal and differentiation to various cell types (Polak and Bishop, 2006). Past studies have demonstrated the application of mesenchymal stem cells from dental pulp (Franca et al., 2020; Rodrigues et al., 2021; Hu et al., 2022) and periodontal ligament (Mishra et al., 2023) within OoC systems to understand host-material interactions and the impact of fluid flow-induced mechanical stress. The potential to derive differentiated cells from pluripotent stem cells can be harnessed in OoC devices for developing patient-specific disease models, drug discovery platforms, and personalized medicine (van der Meer et al., 2013). Further, genetically modified cell lines and stem cells with fluorescent probes provide opportunities for real-time optical assessment of various metabolic responses to biomechanical and chemical cues in OoC devices (Pan et al., 2009).

### 3.6 Matrix considerations

The ECM plays a pivotal role in regulating cellular behavior and function, providing not only structural support but also a reservoir of biochemical and biomechanical signals essential for processes such as differentiation, proliferation, and migration (Theocharis et al., 2016; Frantz et al., 2010). The choice of ECM in OoC systems depends on the type of microenvironment being recapitulated and the factors governing the stability of the ECM proteins in the microsystems. ECM used in OoC systems can be derived from natural, synthetic, or hybrid sources (Mondrinos et al., 2017).

Natural ECM hydrogels, such as collagen and fibrin, are favored for the fabrication of oral mucosa and gingival tissue equivalents owing to their biological relevance and ability to interact with cell surface receptors, promoting cellular activities that are crucial for tissue regeneration and repair. This cell-matrix interactions facilitate cellular activities such as migration, proliferation, and colonization within the hydrogel matrix (Shimshoni et al., 2015). Collagen, particularly types I, II, and III, is the most prevalent ECM protein in connective tissues due to its remarkable tensile strength, making it suitable for OoC applications where stress-bearing tissues are modeled. Collagen hydrogels, particularly type I, is commonly used in the fabrication of full-thickness oral mucosa and gingival tissue equivalents and periodontal OoC systems (Dongari-Bagtzoglou and Kashleva, 2006; Buskermolen et al., 2016; Jennings et al., 2016). However, collagen hydrogels are prone for contraction (Sakulpaptong et al., 2022; Sriram et al., 2015), which poses challenges for long-term *in vitro* studies, as it can hinder the maturation of epithelial barriers and compromise the reliability of drug permeability assays. Oral mucosa-on-chip cultures under flow have also faced similar challenges with collagen matrix contraction restricting long-term culture and maturation of the oral mucosal epithelium, resulting in a thin, immature epithelial barrier and invasion of keratinocytes into the matrix (Rahimi et al., 2018; Ly et al., 2021). Besides affecting epithelial morphogenesis, a non-contracted tissue construct occupying the entire culture chamber is essential to perform reliable drug permeability assays and avoid false-positive spikes in drug permeation kinetics (Macedo et al., 2020; Schmidt et al., 2020). Collagen matrix contraction could be minimized through an optimized fibroblast cell density and collagen hydrogel concentration (Ly et al., 2021; Ly et al., 2022). Alternatively, fibrin-based matrices provide a non-contracting matrix, which have been utilized for the fabrication of gingival connective tissue (Makkar et al., 2023a) and full-thickness gingival equivalents (Muniraj et al., 2023) within OoC systems. In contrast to the fibrillar nature of collagen I, collagen IV forms a mesh-like polymeric structure within the basement membrane (Mak and Mei, 2017), make particularly useful for recapitulating epithelial barrier systems in OoC models (Cramer and Badylak, 2020). While some ECM proteins like fibrin and basement membrane extract (Matrigel^®^) can independently form stable hydrogels, others like laminin, fibronectin, and elastin often require doping within a supporting matrix (Cramer and Badylak, 2020). Other natural ECM hydrogels that could be potentially used for the fabrication of gingival and periodontal tissue equivalents within OoC systems include alginate, nanofibrillar cellulose, hyaluronic acid, chitosan, gelatin and its methacrylated form (GelMA), keratin, decellularised ECM, and silk fibroin.

Synthetic hydrogels like poly (ethylene glycol) (PEG), poly (acrylic acid) (PAA), poly (ethylene oxide) (PEO), poly (vinyl alcohol) (PVA) offer tunable mechanical properties. These hydrogels can be fine-tuned, allowing for controlled assembly initiated by changes in pH, temperature, or light-initiated polymerization making them versatile tools for mimicking ECM in OoC applications (Lee and Sung, 2018). However, these hydrogels often require surface modification or the addition of adhesion peptides to support cell attachment and growth. Hybrid matrices combine the advantages of natural and synthetic components, which provides the biological relevance of natural ECMs while offering the mechanical stability and tunability of synthetic hydrogels. Ultimately, the choice of ECM depends on the specific tissue niche recapitulated and the study goals, focusing on cellular behavior, tissue development, and/or microbial interactions.

The design of OoC systems plays a crucial role in determining the integration of ECM components within the device. In single-channel OoCs used for monolayer culture, the cells and/or bacteria are typically grown directly on the device substrate, which may require functionalization to enhance cellular adhesion. Common substrates, such as glass or PDMS used on the bottom surface of the device can serve as supportive platforms for cell culture (Kutluk et al., 2023). Cells growing on these substrates adapt to the mechanical, topographical, and biochemical cues provided, contributing to the production of their own ECM. Microfluidic channels can be coated or functionalized with cell-adhesive ECM proteins such as collagen, fibronectin, polydopamine, and laminin. These proteins are introduced into the channels and allowed to adhere over a specified period, forming a conducive environment for cell attachment and growth (Ahadian et al., 2018; Huh et al., 2013; Leung et al., 2022; Kutluk et al., 2023). While this coating process may appear straightforward, its success hinges on factors like the wettability of the channel surfaces and the ability to achieve a uniform, stable protein layer that can endure flow conditions within the device. To enhance functionality, additional substrates may be incorporated into the cellular compartments of the OoC. For example, layered production techniques have been utilized to 3D-print and sinter hydroxyapatite (HA)-PDMS composites, which serve as the bottom layer for bone tissue engineering applications on-chip (Tang et al., 2021). This approach replicates the mineralized environment necessary for bone cell differentiation and function.

In multi-channel and multi-chambered OoC devices, the cells could be cultured as monolayers following the strategies utilized for single-channel devices or incorporated within a hydrogel and introduced into the channels or chambers. In horizontally-stacked OoC devices, the ECM hydrogels are typically introduced into the culture chamber through the inlet ports and are compartmentalized within the chamber through phase guides, micropillars or capillary burst valves (Makkar et al., 2023a; Rahimi et al., 2018; Gard et al., 2023). OoC devices used for culture of barrier tissues that allow the air-liquid interface culture are typically designed with vertically-stacked channels and chambers, separated by a porous support membrane. This design allows the fabrication of epithelial barrier tissues including gingival tissues and culture of the tissues at air-liquid interfaces, leading to the induction of apicobasal polarity, epithelial stratification, and differentiation. ECM hydrogels such as collagen and fibrin typically used for these cultures are introduced into the culture chamber either through inlet ports or through removable lids (Kim and Sung, 2024; Jin et al., 2022; Muniraj et al., 2023; Kim and Ingber, 2013; Kim et al., 2012). These ECM hydrogels delivered into the device can be used as coating of the channels and the support membrane, or as cell-laden 3D hydrogels.

## 4 Fluidic approaches to study oral biofilm dynamics

**FIGURE 3:**
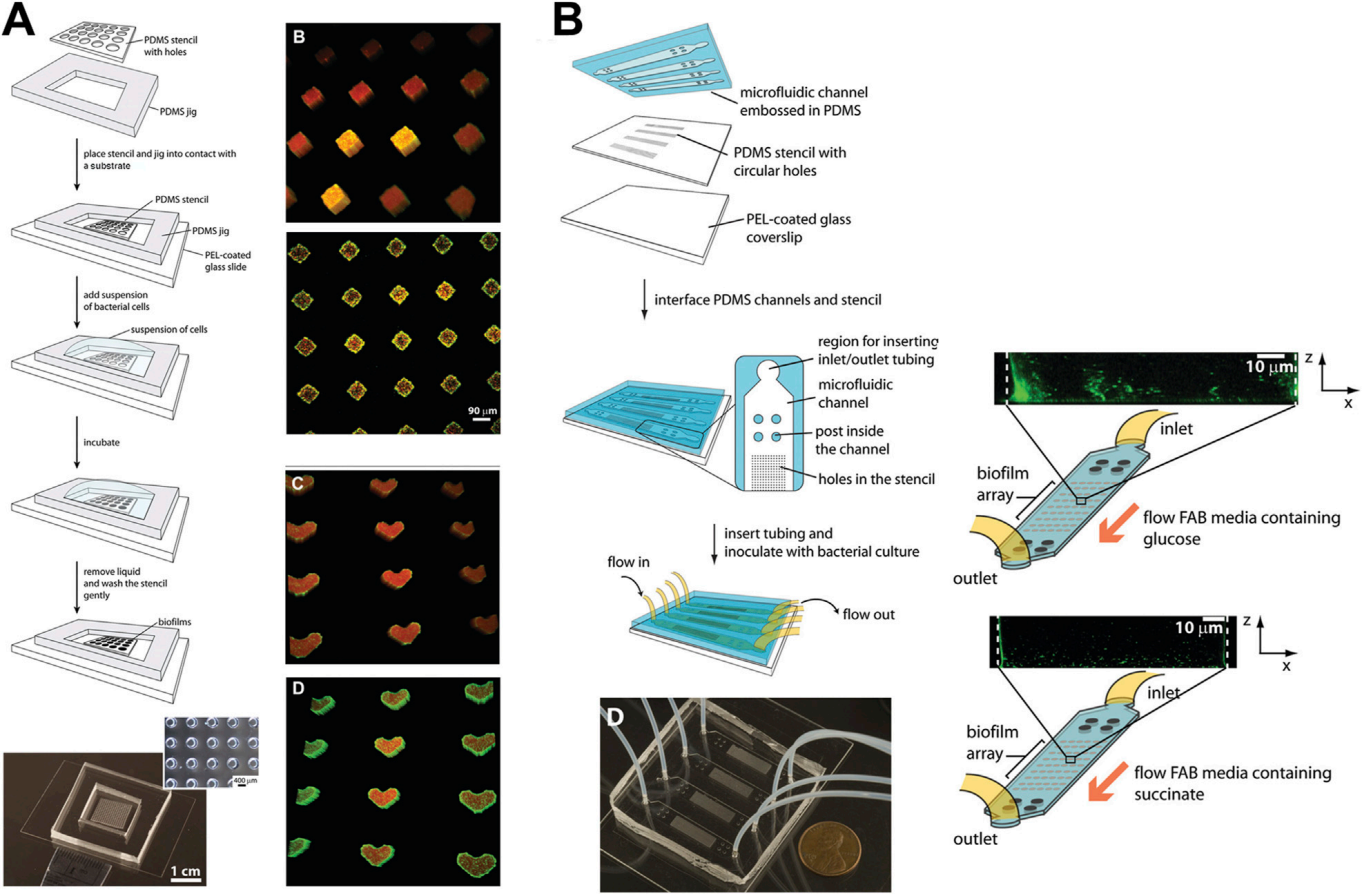
Micropatterning and fluidic devices to study periodontal bacterial biofilms and their dynamics. **(A)** Patterning of bacterial biofilms using an array of micropatterned PDMS stencils. Arrays of *P. aeruginosa* biofilms patterned on diamond and heart-shaped stencils made of various substrates such as polyvinylchloride, polycarbonate, polystyrene, and polyethylene. **(B)** Integration of micropatterned array within a microfluidic device, and culture of biofilm arrays under flow conditions. Figure panels in **(A, B)** are adapted from (Eun and Weibel, 2009) with permission of ©2009 American Chemical Society.

**FIGURE 4:**
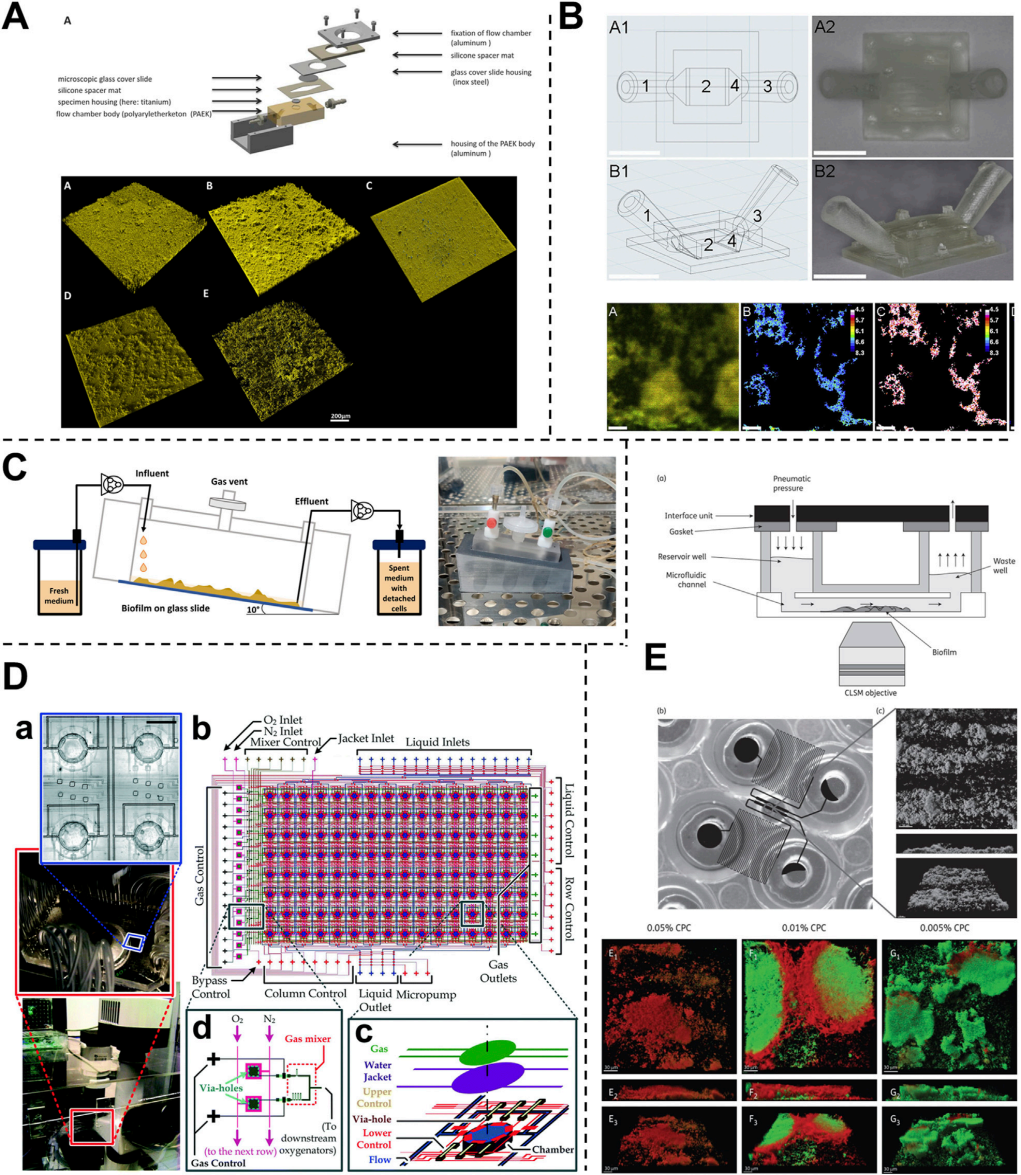
Fluidic devices to study periodontal bacterial biofilms and their dynamics. **(A, B)** Flow cells for the growth of biofilms under a shear-controlled microenvironment, with real-time pH measurement, and assessment of biofilm growth dynamics, and viability. **(C)** Drip-flow reactors with inclined channels enable the formation of biofilms along the direction of liquid flow. **(D)** High-throughput microfluidic device offering control of multiple microbial microenvironmental factors and their impact on spatial distribution, thickness, and viability of oral biofilm colonizers. **(E)** High-throughput BioFlux microfluidic system with in-built pneumatic pressure-driven flow, and its application to assess the anti-bacterial effects of oral-care formulations on plaque biofilms. Figure panels in **(A–E)** are adapted from Rath et al. (2017) under the terms of the CC-BY license, Kristensen et al. (2020) with permission of ©2020 Elsevier, Ghesquiere et al. (2023) under the terms of the CC-BY license, Lam et al. (2016) with permission of ©2016 The Royal Society of Chemistry, and Nance et al. (2013) with permission of ©2013 Oxford University Press respectively.

The complex interplay of various parameters of oral biofilms development and dynamics have been studied using various fluidic systems such as bioreactors, chemostats, flow cells, drip-flow reactors, and microfluidic systems (Wang et al., 2024; Prado et al., 2022) (Figures 3, 4). These fluidic systems have provided the platform to emulate the multi-species interactions, shear flow, gas, and chemical gradients under dynamic conditions offering new insights into oral microbial colonization, adhesion, inter-species interactions, and complex biofilm behaviors.

### 4.1 Bacterial interspecies interactions

Understanding the dynamics within subgingival plaque requires identifying key bacterial species and their potential interactions within the microbial community. In the context of microbiome and biofilm modeling, the role of early colonizers, which establish the initial biofilm matrix, and the subsequent cross-feeding of metabolites are critical for biofilm development and maturation (Marsh and Zaura, 2017; Jakubovics, 2015). In a flow cell system, biofilms cultured separately can be connected via a fluid conduit, allowing culture broth to flow from one chamber to another. This setup enables continuous cultivation and observation of biofilm dynamics over extended periods and provides opportunities to study interspecies interactions and interbacterial cross-feeding relationships. In a study by Zainal-Abidin et al. (2012), red-complex bacteria *T. forsythia*, *T. denticola*, and *P. gingivalis* were simultaneously inoculated in equal volumes into a single-channel flow cell system, which permitted observation of the polymicrobial biofilm for up to 90 h. The study demonstrated that *P. gingivalis* and *T. denticola* became the dominant species in the mature biofilm, while *T. forsythia* diminished over time. Synergistic adaptations between *P. gingivalis* and *T. denticola* were observed, including changes in nutrient sharing and metabolic adjustments. Specifically, *P. gingivalis* exhibited downregulation of glycine catabolism, likely in response to signals from *T. denticola*, enhancing glycine availability for the latter. Concurrently, *T. denticola* upregulated its glycine reductase system, supporting its role in syntrophic interactions within the biofilm. These metabolic changes were associated with enhanced biofilm development and intimate spatial associations between *P. gingivalis* and *T. denticola*. These findings highlighted the mechanical insights into bacterial interactions that are challenging to replicate in traditional microplate platforms. The flow cell system’s ability to facilitate continuous and controlled culture conditions provides a valuable tool for studying the complex interspecies interactions that drive biofilm dynamics, offering insights that are crucial for understanding the microbial ecology of subgingival plaque.

### 4.2 Surface adhesion

Surface adhesion is a critical step in the development of biofilms, distinguishing biofilm-associated bacteria from their planktonic counterparts. In the context of periodontitis, the adhesion of early colonizers to surfaces initiates biofilm formation and maturation by modifying the local microenvironment and creating new adhesion sites for intermediate and late colonizers (Sterzenbach et al., 2020; Love, 2012). In studies modeling oral microbiomes and periodontitis, hydroxyapatite is frequently used as a substrate due to its chemical similarity to tooth enamel. Biofilm adhesion and growth on hydroxyapatite discs under flow conditions have been investigated using flow cells and biofilm bioreactors (Zainal-Abidin et al., 2012; Ramachandra et al., 2024).

Beyond hydroxyapatite, understanding biofilm adhesion on other surfaces, such as human dentin slices, titanium, and zirconium, is equally important (Sterzenbach et al., 2020). Rath and colleagues (Rath et al., 2017) developed a flow cell system to investigate bacterial colonization on implant surfaces. This system recirculated a bacterial suspension containing *S*. *gordonii, S*. *oralis,* and *P*. *gingivalis* at flow rates that simulated natural salivary flow (Figure 4A). Investigating bacterial adhesion to materials like titanium and zirconium is crucial for understanding biofilm formation on implants. Integrating these material surfaces within OoC systems offers valuable opportunities to study the effects of debridement methods under flow conditions, aiding in the development of new debridement tools, such as dental water irrigators, and strategies to enhance biofilm removal. Eun and Weibel (2009) demonstrated the use of microfluidic platforms combined with PDMS stencils to pattern biofilms on geometrically controlled substrates (Figure 3A). This approach enabled precise control over where bacteria could adhere and form biofilms, effectively recreating spatially organized biofilm structures. Further, the potential to study the impact of substrates on biofilm adhesion was demonstrated using diamond or heart-shaped micropatterned biofilm arrays on various substrates including polyvinylchloride, polycarbonate, polystyrene, polyethylene, and stainless steel (Figure 3A). Integrating the micropatterned substrates within a microfluidic device allowed for fine-tuning of fluid dynamics and surface interactions, which are critical factors in the early stages of biofilm development (Figure 3B). By precisely manipulating fluid flow and geometric constraints, it is possible to replicate the conditions bacteria encounter in the oral cavity, thus gaining insights into the initial stages of biofilm formation on dental and implant surfaces. The application of microfluidic platforms in studying biofilm surface adhesion not only provides high-resolution spatial control over biofilm formation but also allows for the investigation of how different surface materials interact with biofilms under physiologically relevant flow conditions.

### 4.3 Shear force and flow rate

Shear forces generated by the movement of saliva within the oral cavity play a crucial role in biofilm development, influencing spatial organization, nutrient uptake, and surface area expansion. Mimicking these liquid shear stresses *in vitro* has been shown to impact biofilm behavior significantly. For instance, *P. gingivalis* biofilms exposed to shear stress exhibited greater resistance to erythromycin compared to those cultured without shear stress, underscoring the importance of replicating such forces in biofilm studies (Maezono et al., 2011). Traditional models like chemostat and biofilm bioreactors, which utilize impellers to generate flow and shear stress, have been used to study oral biofilms. However, these larger systems come with limitations, such as bulkiness, complexity in setup, and the potential for dead zones with low shear stress.

Flow cell bioreactors offer a more refined approach, providing controlled liquid flow through smaller chambers that enable more stable and precise fluid dynamics. Zainal-Abidin et al. (2012), utilized flow cells with a flow rate of 3 mL/h to study the synergistic effects of red complex bacteria, mimicking saliva flow and visualization of biofilm development. These systems offer the advantage of sequential feeding, enabling the investigation of the dynamics of biofilm formation and interaction over time. In another study (Foster and Kolenbrander, 2004), two flow cells containing different bacterial biofilms were linked to study colonization dynamics under a flow rate of 200 μL/min (based on calculated saliva flow rates in the oral cavity). Similarly, Ali Mohammed et al. (2013) employed flow chambers to cultivate *F. nucleatum* and *P. gingivalis* biofilms, using a peristaltic pump to maintain a consistent flow rate of 3.3 mL/h. Drip-flow reactors with inclined channels and gentle continuous flow, enable the formation of biofilms along the direction of liquid flow, closely simulating the conditions within the oral cavity (Figure 4C) (Ghesquiere et al., 2023). The dripping mechanism offers fluid flow with low shear, that could be controlled by a change in the inclination of the biofilm surface. Control over shear stress and flow dynamics in these systems provides a significant advantage, reducing the risk of flow disturbances and ensuring consistent exposure of biofilms to shear forces.

Beyond flow cell systems, microfluidic platforms have emerged as powerful tools for studying biofilms under controlled shear forces. These platforms typically consist of microscale chambers that allow for the simultaneous cultivation of multiple biofilms under highly controlled conditions. The flexible assembly and customization of microfluidic devices enable the recreation of various aspects of the biofilm microenvironment, making them ideal for detailed studies of biofilm formation, behavior, and interactions under specific shear forces. Kristensen et al. (2020) investigated the influence of flow conditions on the pH of dental biofilms (Figure 4B). Their results demonstrated that donor-derived plaque-biofilms maintained under static conditions displayed a significant drop in pH levels. Conversely, those grown under simulated salivary flow conditions exhibited a variable pH throughout the thickness of the biofilm, a phenomenon observed in native oral biofilms. Eun and Weibel (2009) leveraged microfluidic arrays to gain geometric control over biofilm formation, allowing for precise patterning of microbial cells on substrates using PDMS stencils (Figures 3A, B). This setup facilitated the investigation of biofilm growth under different flow conditions, demonstrating that low shear stress (low Reynolds number) and laminar flow in microfluidic channels provide a controlled environment for studying nutrient diffusion and waste removal, which are critical in biofilm dynamics. Similarly, Janakiraman et al. (2009) developed a mathematical model to predict quorum sensing and biofilm growth in closed-loop microfluidic systems, using *Pseudomonas aeruginosa* as a model organism. The model integrated mass transport, shear stress, and biofilm development, addressing the limitations of traditional open system models. Experimental validation in microfluidic chambers showed that the model could effectively predict biofilm thickness and quorum sensing behavior. This work also highlights the utility of microfluidic platforms in simulating the microenvironment of oral biofilms, particularly for exploring how shear forces influence biofilm behaviors and interspecies communication.

In summary, the application of flow cell bioreactors and microfluidic platforms in studying shear forces provides valuable insights into biofilm dynamics. These systems offer precise control over fluid dynamics, making them superior to larger bioreactors in certain applications, particularly when studying the effects of shear stress on biofilm development in the oral cavity.

### 4.4 Controlling gas concentrations

In the subgingival plaque, a diverse array of anaerobic bacterial species thrives, particularly in deep periodontal pockets where low-oxygen environments are prevalent (Celik and Kantarci, 2021). Regulating gas concentrations, particularly oxygen levels, is crucial in cultivating oral biofilms, especially those involving obligate anaerobic pathogens associated with periodontal diseases, such as *P. gingivalis*, *T*. *forsythia*, and *T*. *denticola* (Celik and Kantarci, 2021). Traditional methods for studying these anaerobic bacteria involve placing the culture plates in anaerobic chambers with a gas mixture containing 10% H_2_, 10% CO_2_, and balanced N_2_. While effective, these chambers provide a uniform gas environment, which may not be ideal for multi-species or host-bacteria co-cultures that require differential oxygen conditions.

Chemostat systems have been utilized to create controlled gaseous environments for studying the effects of oxygen on oral microbial communities. In Bradshaw et al.'s study (Bradshaw et al., 1996), two chemostats were connected, with one chamber maintaining anaerobic conditions (aerated with CO_2_ and N_2_) and the second receiving oxygen-rich air. This setup allowed the investigation of the impact of oxygen on oral microbes like *Neisseria subflava* and *streptococci*. Interestingly, even after transitioning the mixed culture from the anaerobic to the oxygenated chemostat, anaerobic species continued to thrive, indicating a certain level of oxygen tolerance. Similarly, Diaz et al. (2002) employed a chemostat to co-culture *P. gingivalis* and *F. nucleatum*, exposing them to gradually increasing oxygen levels. Their findings revealed that *P. gingivalis*, which is typically sensitive to oxygen, exhibited enhanced survival in the presence of *F. nucleatum* under oxygen-rich conditions, demonstrating the protective effects of microbial interactions.

Microfluidic platforms offer advanced capabilities for precisely controlling oxygen levels across different microenvironments. These systems utilize microchannel networks to spatially distribute oxygen, enabling the cultivation of biofilms under tightly regulated conditions. For instance, Lam et al., developed a microfluidic “artificial teeth” device with 128 incubation chambers, each independently controllable for nutrient and gas concentrations (Lam et al., 2016). This high-throughput platform facilitated the regulation of various microenvironmental factors, including dissolved gas concentrations, nutrient delivery, and microbial seeding density (Figure 4D). The platform also enabled the quantitative assessment of biofilm characteristics, including thickness, viable-dead cell ratios, and species distribution under different dissolved gas conditions and sucrose concentrations.

A key challenge in modeling modelling periodontal host-microbe interactions *in vitro* has been the long-term co-culture of host cells and obligate anaerobes, given their distinct microenvironmental requirements. The predominance of obligate anaerobes within the dysbiotic periodontal microbiota poses an extremely stressful environment for the host cells. To overcome these challenges, inspirations can be drawn from gut-on-chip systems, which have paved the way towards emulating the oxic-anoxic interface or gradients between the host and bacterial compartments. For instance, gut-on-chip systems fabricated using PDMS have been used to create oxic-anoxic interface (Shin et al., 2019). Similarly, thermoplastic multi-compartment devices have been designed to perfuse the cellular and bacterial compartments with media having defined oxygen concentrations (Shah et al., 2016). Further, the incorporation of oxygen probes within gut-on-chip systems have provided real-time monitoring of oxygen concentrations within the respective compartments (Jalili-Firoozinezhad et al., 2019). By refining culture conditions through OoC devices with design features that enable differential oxygen gradients, our understanding and recapitulation of these complex interactions *in vitro* can be significantly enhanced.

The liquid environment plays a crucial role in the development and behavior of oral biofilms, with saliva providing the necessary nutrients, fluid dynamics, and chemical milieu. To simulate these conditions *in vitro*, various systems have been developed that allow precise control over the liquid environment, influencing biofilm growth and structure. Chemostats, which maintain a continuous flow of nutrients and control the pH, have been used to mimic the oral environment’s dynamic conditions. Zilm and Rogers (2007) utilized a chemostat-based biofilm culture system to study *F. nucleatum*, a key pathogen in oral biofilms. By carefully controlling the pH of the culture medium, they observed that increasing the pH led to significant changes in cell morphology and biofilm growth, peaking at a pH of 8.2.

Microfluidic platforms have emerged as tools for controlling the liquid environment in biofilm studies, offering several advantages over larger systems like chemostat and flow cells. These platforms use microlitre-scale chambers, which significantly reduce the volume of saliva or other fluids needed for biofilm cultures. For instance, Nance et al. (2013) used the BioFlux microfluidic system to inoculate and culture biofilms in sterilized saliva, assessing antibacterial effects within small, controlled environments (Figure 4E). The design of the BioFlux microfluidic system is microscale (48 wells, each 70 μm deep and 370 μm wide), minimizes the amount of saliva needed, and allows for precise manipulation of the fluid flow. The 48-well format allows for the culture of multiple biofilms under different conditions and facilitates high-throughput studies. Microfluidic systems can be utilized to study the responses of biofilms under various liquid environments, including fluctuations in nutrient availability and chemical composition. The small size of microfluidic channels can allow for the generation of gradients, where biofilms can be exposed to gradually changing conditions that closely mimic the *in vivo* environment.

## 5 Fluidic systems to emulate dental and periodontal host-microbe-material studies

**FIGURE 5:**
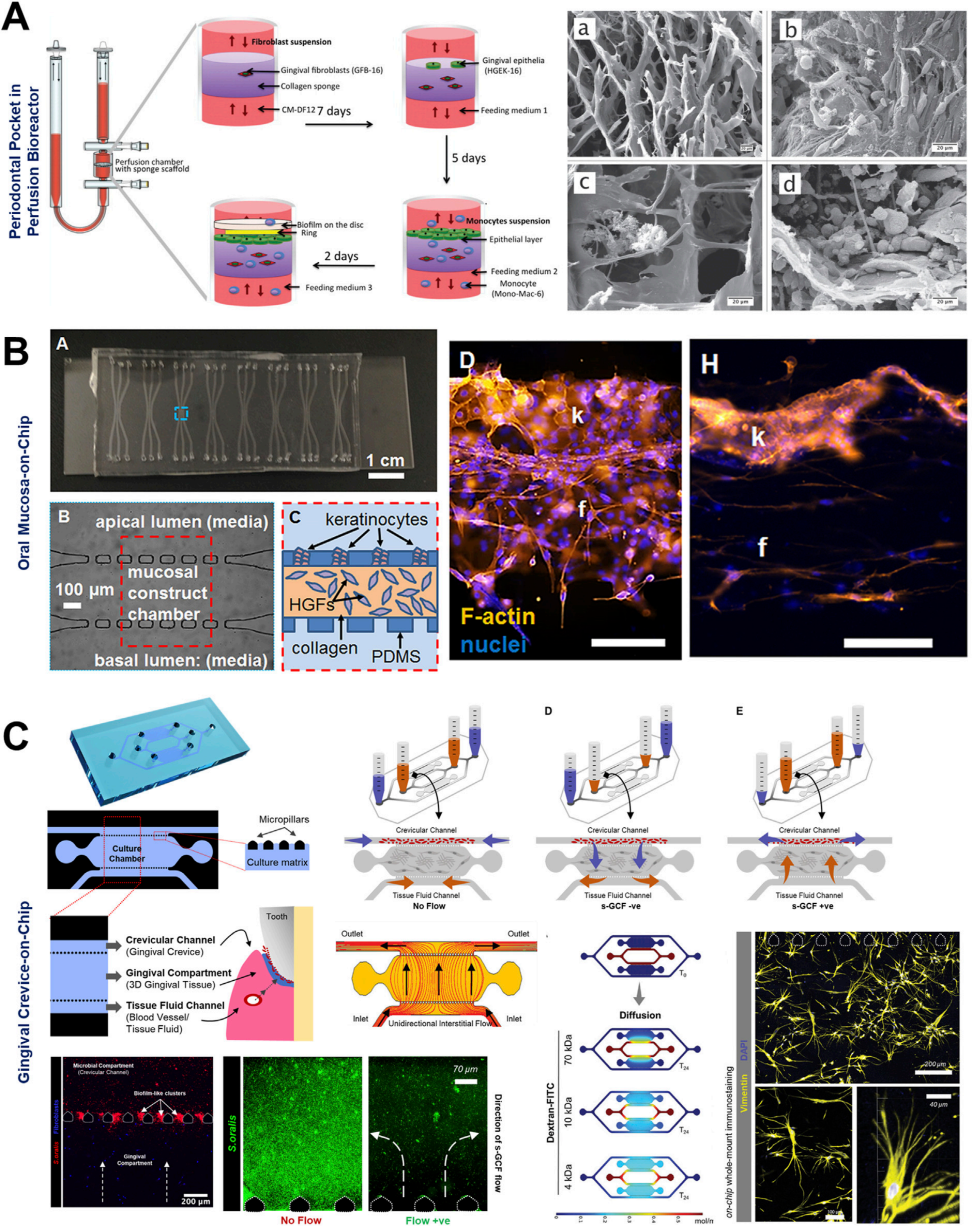
Organ-on-Chip design configurations to emulate interstitial fluid flow and long-term host-microbe interactions. **(A)** Perfusion bioreactor-based fluidic device to simulate periodontal pocket and study the interaction between gingival wall, immune cells, and complex periodontal biofilms under perfused conditions. Microfabricated oral mucosa-on-chip **(B)** and gingival crevice-on-chip **(C)** devices with horizontally-stacked configuration and their application for host-microbe interactions. Rectangular or pentagonal posts compartmentalize the culture chamber from adjoining bacterial and media channels. Appropriate inter-post distance and chamber height allow the loading and confinement of cell-laden hydrogels within the chamber. The adjoining channels allow the seeding of other cell types, oral microbes, biomaterials, and drugs. The use of hydrostatic pressure gradient between reservoirs **(C)** can be used to emulate interstitial and gingival crevicular fluid flow representative of health and diseased states. Figure panels in **(A–C)** are adapted from Bao et al. (2015b) under the terms of the CC-BY license, Rahimi et al. (2018) with permission of AIP Publishing, and Makkar et al. (2023a) with permission of ©2022 Wiley‐VCH GmbH respectively.

**FIGURE 6:**
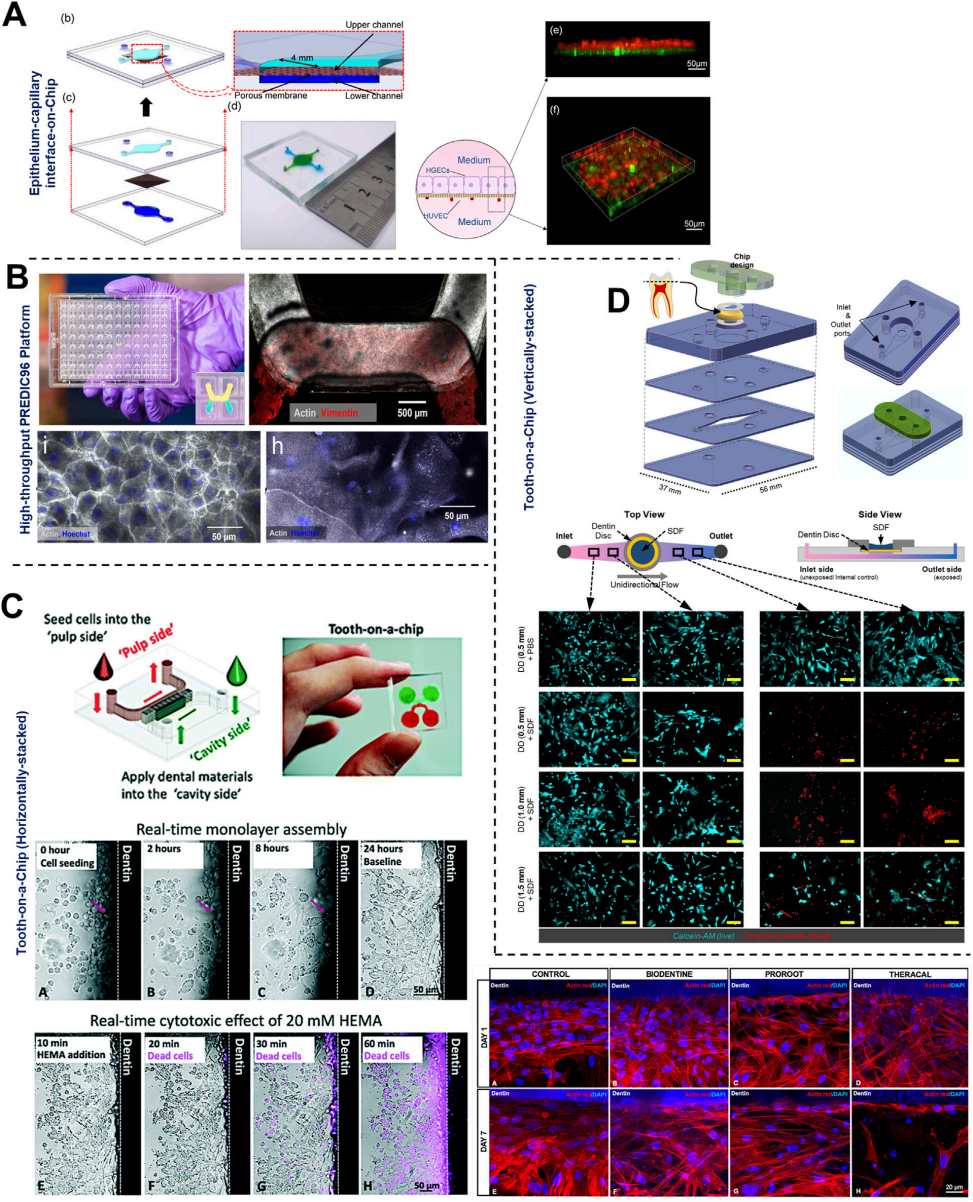
Organ-on-Chip design configurations to emulate barrier integrity and host-microbe-material interactions. **(A, B)** Vertically-stacked OoC device configurations with a porous support membrane dividing the culture chambers and their application for multicellular cellular culture to emulate epithelial and endothelial barrier under air-liquid interface and active media flow. These devices can be fabricated in **(B)** multi-device configuration containing 96 devices in a single plate format for long-term culture of cells and high-throughput drug screening applications. Tooth-on-chip fluidic devices with horizontal **(C)** and vertical **(D)** stacked configurations to recapitulate dental cavity-dentin-pulp interface, dentin barrier, and its application to study dental biomaterial toxicity. These devices enable the incorporation of *ex vivo* human dentin slices separating the pulp cells from the biomaterials and bacteria applied on the tooth. These platforms have been used to study biocompatibility **(C, D)** and antibacterial properties **(C)** of dental biomaterials. Figure panels in **(A, B)**, and **(D)** are adapted from Jin et al. (2022) under the terms of the CC-BY license, Gard et al. (2023) under the terms of the CC-BY license, and Hu et al. (2022) with permission of ©2022 The Academy of Dental Materials respectively. Figure panels in **(C)** is adapted from (Franca et al., 2020) with permission of ©2020 Royal Society of Chemistry, and from Rodrigues et al. (2021) with permission of ©2021 Sage Publications.

**FIGURE 7:**
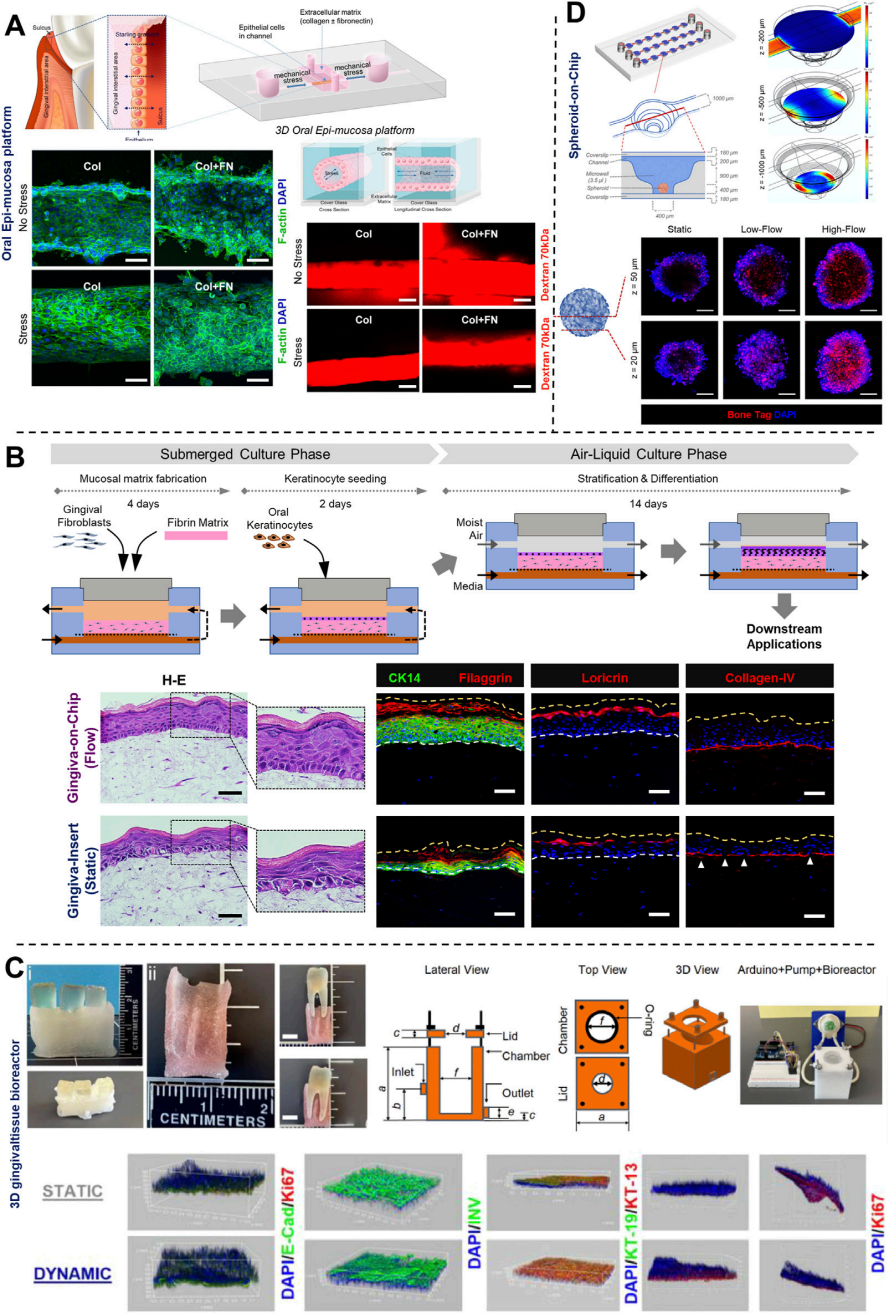
Understanding the impact of mechanical forces, fluid flow, and associated shear stress on periodontal health using OoC systems. **(A)** 3D oral epi-mucosa device demonstrates the impact of mechanical forces and matrix stiffness on oral epithelial barrier function. **(B)** Gingiva-on-Chip millifluidic device for culture of full-thickness gingival equivalents under air-liquid interface. This platform demonstrates the impact of fluid flow on epithelial morphogenesis, maturation, and barrier function, and its integration for downstream application to emulate the mechanical forces associated with oral-care formulations and associated host-material responses. **(C)** 3D printed tooth-periodontal interface integrated within a custom-designed bioreactor for the culture of gingival cells, recapitulate salivary flow dynamics, oxygen gradient, and study the impact on epithelial morphogenesis, barrier function, and innate immune response of gingival tissues to periodontal pathogens. **(D)** Culture of periodontal mesenchymal stromal cell-derived spheroids on-chip under differential fluid shear stresses and its spatiotemporal impact on osteogenic differentiation. Figure panels in **(A–D)** are adapted from (Lee et al., 2023) under the terms of the CC-BY license, (Muniraj et al., 2023) under the terms of the CC-BY license, (Adelfio et al., 2023) with permission of ©2023 Acta Materialia Inc, and (Mishra et al., 2023) with permission of ©2023 The Royal Society of Chemistry respectively.

The application of microfluidic OoC systems to replicate the host-microbe-material interface, tissue fluid dynamics, and shear stress has gained significant traction in the last decade, thereby offering new insights into host-microbe and host-material interactions in health and disease states of the dental and periodontal tissues (Figures 5–7).

### 5.1 Simulation of host-microbial interface, fluid flow, and host defense mechanisms

To understand the complex interactions between gingival tissues and microbial biofilms, Bostanci and colleagues (Bao et al., 2015a; Bao et al., 2015b), developed an *in vitro* model of a periodontal pocket utilizing a perfusion bioreactor (Figure 5A). This model comprised a scaffold seeded with fibroblasts and keratinocytes representing the wall of a periodontal pocket, and a complex multi-species biofilm of red-complex bacteria cultured on hydroxyapatite discs, simulating the microbial component representative of periodontal disease. Despite its relatively large size, this perfusion reactor-based system established the foundations for fluidic-based systems designed to replicate the intricate host-microbe interactions *in vitro*.

OoC devices designed to mimic oral mucosal and gingival tissues have demonstrated the potential for long-term co-culture of gingival tissues and oral bacteria, a common limitation associated with static culture systems (Makkar et al., 2023a; Adelfio et al., 2023; Jin et al., 2022; Rahimi et al., 2018). These devices, incorporate features like microchambers and microchannels for the compartmentalized culture of host cells and microbes as demonstrated in gingival crevice-on-chip (Makkar et al., 2023a), epithelium-capillary interface-on-a-chip (Jin et al., 2022), and oral mucosa-on-chip (Rahimi et al., 2018). Under dynamic flow conditions, these systems have demonstrated long-term host-microbe co-culture, simulated the protective effects of GCF flow, and the modulation of barrier integrity. Raub and colleagues (Rahimi et al., 2018) developed a 3-channel oral mucosa-on-a-chip, which incorporated keratinocytes seeded onto a microchannel beside a microchamber loaded with gingival fibroblast-populated collagen hydrogel (Figure 5B). This system was employed to explore responses to dental materials and oral bacteria (Rahimi et al., 2018; Ly et al., 2021), model oral mucositis after radiation and chemotherapy (Ly et al., 2022), and investigate potential therapeutic targets for oral mucositis recovery (Ly et al., 2024). Through this series of work, they also demonstrated the potential approaches to impair and recover oral mucosal barrier function.

GCF which originates from interstitial fluid flow through gingival connective tissue serves a protective role by washing away bacteria and toxins, delivering immune cells and antimicrobial agents to combat plaque bacteria, and maintaining tissue health (Fatima et al., 2021). Makkar et al. (2023a), designed a gingival crevice-on-chip with a hexagonal microchamber sandwiched between two microchannels to emulate GCF and interstitial fluid flow through the gingival crevice and connective tissue respectively (Figure 5C). Employing computational fluid dynamics and fluorescence recovery after photobleaching (FRAP)-based assay, they investigated the kinetics of macromolecular diffusion through the gingival connective tissue. These findings provide insights into the macromolecular perfusion kinetics representative of healthy and inflamed states. Further, the simulation of GCF flow *in vitro* has provided unique opportunities to demonstrate its protective effects including mechanical clearance of bacteria and modulation of innate immune responses against periodontal pathogens (Makkar et al., 2023a). Similarly, Jin et al. (2022), demonstrated recovery of barrier integrity in gingival tissues exposed to TNF-α and LPS on an epithelium-capillary interface-on-a-chip (Figure 6A). Further, they demonstrated the potential to assess selective permeability to different macromolecules and endothelial barrier integrity. Similarly, Gard et al. (2023) demonstrated the fabrication of a vascularized gingival tissue using a triculture of keratinocytes, fibroblasts, and microvascular endothelial cells within a PREDICT96 microfluidic platform (Figure 6B). This platform allows for high-throughput experimentation with programmable fluid flow and integrated sensing capabilities microfluidic plate integrated with an automated pump. The gingival tissue exhibited stratified architecture with mucosal barrier formation that was stable over a 20-day culture period. Inflammatory state induced using a cocktail of inflammatory cytokines (TNF-α and IL-1β) resulted in a decrease in TEER and an increase in the secretion of pro-inflammatory markers such as PGE2, MIP-3α, IL-10, and IFN-γ. Importantly, the model also demonstrated tissue recovery following the removal of the inflammatory stimulus and the application of small-molecule inhibitors. This recovery was evidenced by the restoration of TEER values and a reduction in inflammatory marker levels, highlighting the potential for evaluating the efficacy of therapeutic agents aimed at mitigating inflammation and promoting tissue healing. Overall, these studies highlight the potential of OoC-based models to emulate the host tissue-microbe interface of oral and gingival tissues under flow conditions and enable long-term host-microbe interactions. These capabilities enable to study gingival inflammation *in vitro*, and provide the opportunity for the identification and screening of new therapeutics for periodontal disease.

OoC systems have also provided new insights into the interactions of biomaterials and microbes on dental tissues and dental caries. Franca et al. (2020) developed the tooth-on-a-chip model, a horizontally-stacked 3-chamber device that enabled the observation of dental pulp responses to dental materials applied onto the dentin surface (Figure 6C). This pioneering model revealed significant insights into the cytotoxic effects of dental materials on dental pulp, as well as their influence on gelatinolytic activity within a hybrid layer. Using the tooth-on-chip platform, Rodrigues et al. (2021) investigated the effects of pulp capping materials on dental pulp stem cells. Their findings highlighted the ability of ProRoot (a calcium silicate-based pulp capping material) to promote TGF-β release from dentin slices, and inhibit *Streptococcus mutans* biofilm formation, demonstrating the platform’s effectiveness in assessing dental materials’ biocompatibility and antimicrobial properties. Similarly, Hu et al. (2022), developed a vertically-stacked tooth-on-chip device to study the cytotoxic effects of silver diamine fluoride (SDF) on dental pulp cells (Figure 6D). Their findings demonstrated that thinner dentin barriers (≤1.0 mm) allowed significant SDF penetration, leading to substantial death of dental pulp cells. Further, their design incorporated the inlet channel to serve as an internal control, providing a direct comparison against the outlet channel that was exposed to SDF. This configuration enabled the observation of spatial differences in cell viability, with the inlet side (unexposed to SDF) showing high viability, while the outlet side (exposed to SDF) exhibited substantial cell death. These tooth-on-chip models have significantly advanced the ability to visualize and quantify the effects of dental materials on the dentin-pulp complex in real-time, thus providing valuable insights that can drive the development of more effective and bioactive dental materials.

### 5.2 Simulation of mechanical and shear stress

The gingiva and periodontal ligament are routinely subjected to mechanical forces and shear stress. These arise from salivary flow and daily activities such as mastication, brushing, and the use of oral-care products. To better understand the impact of these forces on gingival/periodontal health, various OoC systems have been developed to replicate the mechanical environment of the oral cavity and study their effects on epithelial integrity and tissue response (Figure 7).

Using a 3D oral epi-mucosa platform, Lee et al. (2023), demonstrated that collagen matrices with an intermediate stiffness (30 Pa) were optimal for maintaining barrier integrity. In contrast, matrices that were either softer (10 Pa) or significantly stiffer (120 Pa) led to a compromised barrier function, emphasizing the importance of mechanical properties in gingival tissue health (Figure 7A). In another study, Muniraj et al. (2023) presented a microfluidic gingiva-on-chip platform designed to biofabricate full-thickness gingival tissue and ulcerated equivalents (Figure 7B). This platform demonstrated that dynamic flow conditions enhanced epithelial morphogenesis and improved barrier functionality, offering a more physiologically relevant environment compared to static culture systems. Additionally, the platform was employed to study the mechanical effects of mouthrinse on intact and ulcerated gingival tissues. Their findings indicated that dynamic exposure to these products resulted in more pronounced tissue disruption and cytotoxic effects than static exposure, underscoring the significance of mechanical stress in oral tissue response and evaluation of oral-care formulations. Similarly, Adelfio et al. (2023) engineered a dynamic gingival tissue model cultured within a custom bioreactor, through which a media mimicking the composition, viscosity, and non-Newtonian behavior of human saliva was flown (Figure 7C). Their findings demonstrated that the physiological shear stress induced by salivary flow enhanced epithelial barrier function. Further, the salivary flow supported the long-term stability of gingival tissues when exposed to *P. gingivalis* LPS. Using a microfluidic spheroid-on-chip platform, Mishra et al. (2023), investigated the impact of fluid shear stress on spheroid cultures of periodontal ligament stem cells (PDLSCs) (Figure 7D). Their findings demonstrated that fluid flow conditions, in particular high fluid shear stress (360 µPa), significantly enhanced the viability and osteogenic differentiation of PDLSC spheroids. Further, the study observed spatial differences in osteogenic differentiation within the spheroids, influenced by the fluid flow. High-fluid shear stress conditions resulted in more uniform and enhanced osteogenic differentiation throughout the spheroid, while static conditions showed osteodifferentiation activity restricted to the periphery of the spheroid.

### 5.3 On-chip and off-chip assays

**FIGURE 8:**
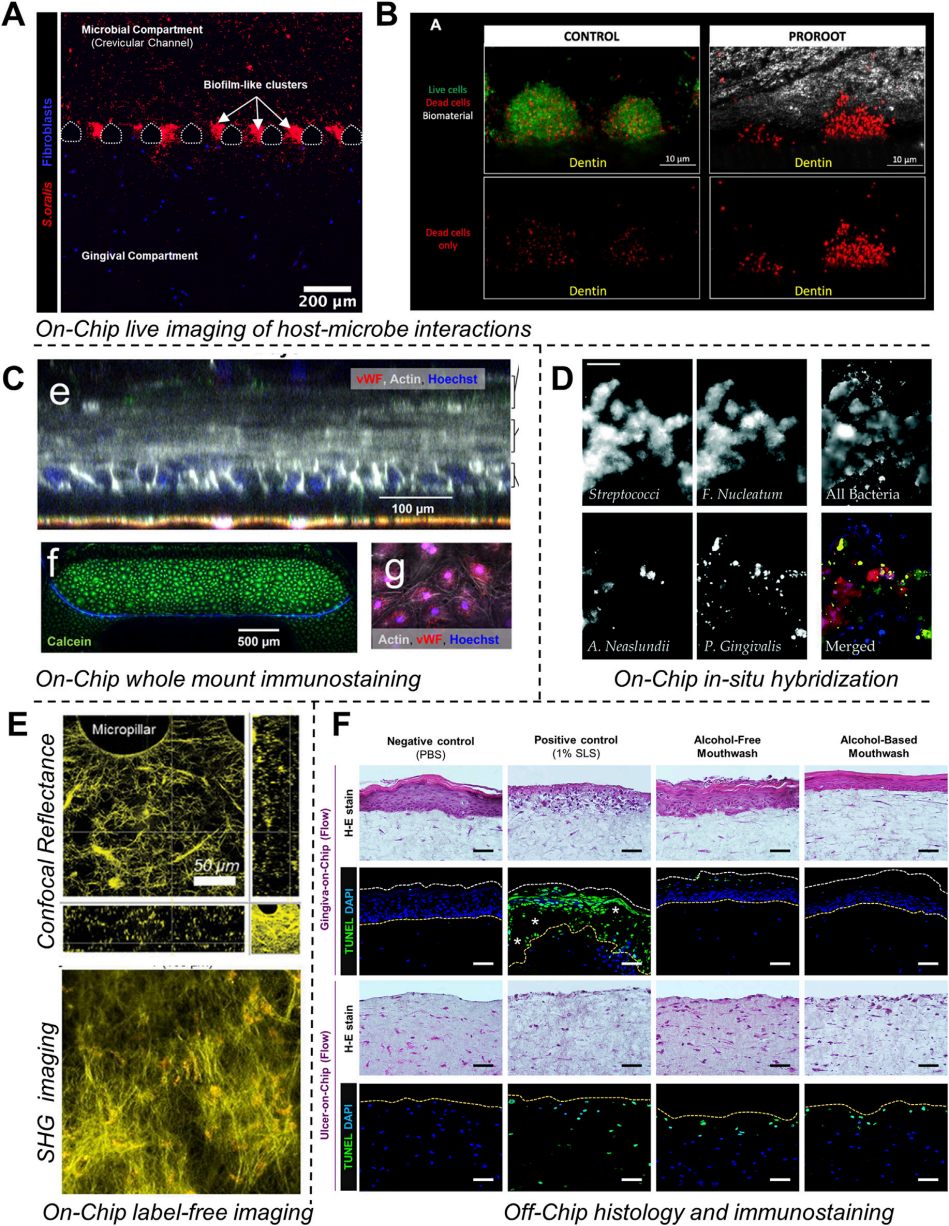
On-chip and off-chip readouts on OoC devices. **(A, B)** On-chip visualization of spatiotemporal oral host-microbe interactions and impact of antibacterial properties of dental biomaterials. **(C)** Visualization of viability and morphological characteristics of the epithelial and endothelial interface using on-chip whole-mount immunostaining and confocal microscopy. **(D)** Visualization of microbial biofilms using fluorescence *in-situ* hybridization of bacteria-specific probes on a high-throughput microfluidic device. **(E)** On-chip label-free and non-invasive visualization of cellular and matrix elements of tissue equivalents using confocal reflectance microscopy and second harmonic generation imaging. **(F)** Off-chip visualization of viability and morphological characteristics following culture and exposure to external agents following histology and immunostaining of harvested tissues from gingiva-on-chip and ulcer-on-chip equivalents. Figure panels in **(A–D, F)** are adapted from Makkar et al. (2023a) with permission of ©2022 Wiley‐VCH GmbH, Rodrigues et al. (2021) with permission of ©2021, Sage Publications, Gard et al. (2023) under the terms of the CC-BY license, Lam et al. (2016) with permission of ©2016 The Royal Society of Chemistry, Muniraj et al. (2023) under the terms of the CC-BY license respectively. Figure panels in **(E)** is adapted from Makkar et al. (2023a) with permission of ©2022 Wiley‐VCH GmbH, Sriram et al. (2018) under the terms of the CC-BY license.

Various on-chip and off-chip assays have been used to assess the interactions between host tissues, microbes, and materials (Junaid et al., 2017) (Figure 8). The transparency of microfluidic devices, design configuration, coupled with cellular labeling and imaging modalities, enables real-time visualization of host cells, ECM, and microbial communities within the chip. For instance, horizontally-stacked microfluidic channel configuration enables on-chip visualization and tracking of live host cells and microbes (Makkar et al., 2023a) (Figures 8A, B). Additionally, immunostaining and whole-mount confocal imaging can be employed to analyze cellular organization and differentiation (Figure 8C). Markers such as F-actin (Jin et al., 2022; Rahimi et al., 2018) and vimentin (Makkar et al., 2023a) are used to visualize fibroblasts, while keratinocyte stratification and differentiation can be monitored using cytokeratins and barrier proteins (Adelfio et al., 2023; Muniraj et al., 2023; Ly et al., 2024), offering insights into tissue development. Similarly, endothelial cells and microvasculature can be visualized using whole-mount imaging of tissue equivalents stained with endothelial markers like CD31, VE-cadherin, vWF, collagen-IV, and laminin-V (Jin et al., 2022) (Figure 8C). Bacterial populations within fluidic devices can be monitored in real-time using labels like SYTO 9 (Rodrigues et al., 2021; Rath et al., 2017) and peptidoglycans (Makkar et al., 2023a) or through endpoint assays like fluorescence *in situ* hybridization (FISH) (Lam et al., 2016) (Figure 8D)**.** Advances in label-free imaging techniques, including confocal reflectance microscopy, multiphoton microscopy, and second harmonic generation, have further enhanced the ability to study live tissue constructs without the need for exogenous labels. 3D microscopy using confocal reflectance and second harmonic generation enables the label-free visualization of collagen and other ECM components and their organization within live and fixed tissue samples (Makkar et al., 2023a; Makkar et al., 2023b; Sriram et al., 2020; Atkuru et al., 2021) (Figure 8E). Similarly, two-photon excited fluorescence from endogenous fluorophores like NADH and FAD allows for non-invasive imaging of keratinocytes, fibroblasts, and endothelial cells (Sriram et al., 2018; Sriram et al., 2020; Lee et al., 2022; Martínez-Ojeda et al., 2020). Integrating biosensing technologies within microfluidic systems has further expanded the scope of real-time monitoring (Figures 4D, 6B). Immunosensors can be incorporated to detect and quantify inflammatory mediators such as interleukin-6 and tumor necrosis factor-α (Kaur et al., 2018; Zhou et al., 2015; Riahi et al., 2016), while oxygen-sensing probes can provide insights on oxygen gradients (Shah et al., 2016; Jalili-Firoozinezhad et al., 2019) crucial for replicating the aerobic-anaerobic gradient between host tissue and periodontal pathogens. Similarly, integrating trans-epithelial electrical resistance (TEER) sensors on-chip can enable real-time, label-free monitoring of epithelial barrier integrity following exposure to periodontal pathogens or their surrogates (Shah et al., 2016).

Off-chip assays using the media collected from the outlet ports of fluidic devices can be utilized to complement on-chip assays. For instance, lactate dehydrogenase (LDH) assays provide a means to assess cell viability and cytotoxicity over time by measuring LDH levels in the culture media (Makkar et al., 2023a). Tissue equivalents cultured on-chip can be retrieved, processed, and immunostained for key cellular differentiation markers, viability and histometric analysis (Muniraj et al., 2023) (Figure 8F). Similarly, enzyme-linked immunosorbent assays (ELISA) can be used to quantify the secretion kinetics of pro- and anti-inflammatory cytokines from collected media (Makkar et al., 2023a; Jin et al., 2022; Rahimi et al., 2018), offering critical insights into immune responses, periodontal pathogenesis, and healing processes.

## 6 Challenges, future directions and conclusions

The integration of millifluidic and microfluidic technologies into periodontal research represents a significant advancement, providing physiologically relevant models to study the dynamics of oral biofilms, host-microbe and host-material interactions. Despite these advancements, several challenges and opportunities for future research remain. A primary area for development is the enhancement of the cellular, tissue, and bacterial complexity to better mimic the native oral environment. This includes the incorporation of additional cellular components such as immune cells, which play a critical role in periodontal disease progression, as well as refinement of ECM hydrogels to reflect the dynamic processes of tissue remodeling and degradation during disease states. Additionally, incorporating vasculature and immune cells provides opportunities to study bacterial invasion, dissemination, and host defense mechanisms (Alonso-Roman et al., 2024; Maharjan et al., 2020). Furthermore, incorporation of multi-species biofilms and validation using plaque samples from health and disease sites is pivotal towards increasing the physiological relevance of the periodontal OoC systems.

Exploring complex host-microbe interaction studies using these devices can aid in understanding the mechanistic pathways of immune subversion by oral microbes, kay area that needs to be explored. The gingival mucosa and periodontal tissues support a dynamic interplay between host immune mechanisms and bacterial strategies, where pathogenic bacteria can manipulate host immune responses to evade clearance and sustain local inflammation. For instance, *P. gingivalis*, *T. forsythia*, and *P. intermedia* can protect themselves and nearby bacteria from being cleared by the human immune system by blocking and degrading complement activation (Popadiak et al., 2007). Bacterial proteases, such as gingipains produced by *P. gingivalis*, *T. forsythia*, and *P. intermedia*, work together to inhibit complement activation, offering additional protection to complement-sensitive bystander bacteria (Jusko et al., 2012). Meanwhile, *T. denticola* prevents the production of human β-defensins by gingival epithelial cells in response to *F. nucleatum* (Shin et al., 2013). It also blocks the fusion of internalized *F. nucleatum* with lysosomes and suppresses the production of reactive oxygen species, thus inhibiting TLR signaling that regulates β-defensin expression. Mimicking these microbe-immune cross-talks and immune evasion mechanisms using the OoC technologies as well as integration with other multi-modal omics technologies may serve as a powerful strategy to understand host-microbe interactions, identify biomarkers, and develop novel periodontal therapeutic strategies (Hajishengallis, 2015).

The scalability and standardization of OoC platforms are crucial for broader adoption. While current fluidic systems provide detailed insights at the micro-scale, there is a pressing need for platforms capable of handling larger sample sizes and enabling high-throughput analysis. This will require advancements in fabrication techniques, and the development of robust, user-friendly interfaces that can be easily integrated into existing research workflows (Zhang and Radisic, 2017). Another significant direction for future research is the integration of real-time monitoring and biosensing technologies (Junaid et al., 2017). Progress has been made in incorporating sensors for monitoring parameters such as oxygen levels and cytokine secretion in gut-on-chip systems (Kim and Ingber, 2013; Kim et al., 2012; Shah et al., 2016). The development of more sensitive and specific biosensors could facilitate real-time detection of a wider range of biochemical signals and biomarkers, providing deeper insights into the complex interactions between host tissues and microbial communities.

Future research can draw valuable insights from gut-on-chip systems (Kim and Ingber, 2013; Kim et al., 2012; Shah et al., 2016), which effectively model the aerobic-anaerobic interface between host tissues and microbial biofilms. Adapting these approaches to periodontal models can better emulate similar interfaces within the oral cavity. Gut-on-chip systems have been utilized to study probiotic-pathogen interactions, demonstrating the capability of OoC platforms to rapidly screen multiple probiotics strains (Wu et al., 2024), and investigate mechanisms for ameliorating gut inflammation (Min et al., 2022). Applying these insights to the study of periodontal pathogen-probiotic interactions could unlock significant potential to screening novel therapeutic adjuvants for the treatment of periodontal dysbiosis (Canut-Delgado et al., 2021). Furthermore, integrating periodontal fluidic systems with other OoC models could yield deeper insights into the systemic effects of periodontal diseases, particularly in understanding how oral biofilms contribute to conditions such as cardiovascular disease, diabetes, and gut health. By addressing these challenges and embarking on these opportunities, we can advance towards developing robust, predictive models that enhance our understanding of periodontal diseases, pave the way for novel therapeutic strategies, and provide a platform to explore their links to systemic health and overall wellbeing.

One of the primary goals of OoC models is to accurately replicate human physiological responses *in vitro*, aiming to provide outputs that align closely with clinical diagnostics. Establishing a clear correlation between OoC data and clinical measurements remains challenging, primarily due to differences in measurement methods and techniques, which complicate the integration of OoC data with clinical insights to accurately reflect real-world organ function and disease states. Incorporating control loop engineering techniques into OoCs will enable real-time manipulation of readouts, facilltating a more accurate simulation of the dynamic and living nature of a patient (Capulli et al., 2014). As OoCs are inherently simplified models of organs, their readouts represent only a subset of the full spectrum of organ functions assessed in clinical settings. To bridge this gap, it is essential to continually refine design approaches, select cost-effective materials, optimize manufacturing processes, scale up production, and conduct clinical correlation studies to enhance the accessibility and physiological relevance of OoC systems (Ingber, 2020).

In conclusion, the integration of OoC technologies into periodontal research holds remarkable potential for advancing our understanding of host-microbe interactions and the pathogenesis of periodontal diseases. By addressing the present challenges in system complexity, scalability, and real-time monitoring, and drawing inspiration from related technologies like gut-on-chip systems, these platforms can be further developed to provide comprehensive insights into periodontal health and its systemic implications. As these innovations progress, they are poised to not only improve therapeutic strategies but also contribute significantly to the broader field of personalized medicine, ultimately enhancing both oral and overall health outcomes.
